# Diversity of enzymes for exopolysaccharide synthesis in the fructophilic honeybee symbiont *Apilactobacillus kunkeei*

**DOI:** 10.1186/s12866-025-04680-3

**Published:** 2026-01-12

**Authors:** Marina Mota-Merlo, Julia E. Pedersen, Siv G. E. Andersson

**Affiliations:** Department of Cell and Molecular Biology, Molecular Evolution, Science for Life Laboratory, Biomedical Centre, Uppsala University, Uppsala, Sweden

## Abstract

**Background:**

*Apilactobacillus kunkeei* is a fructophilic lactic acid bacterium adapted to honeybees, their food sources and products. These bacteria synthesize exopolysaccharides thought to promote host colonization and protection against toxic compounds and stressful conditions. Homopolysaccharides consisting of glucose residues are synthesized by enzymes in the glycoside hydrolase family 70 (GH70), whereas polysaccharides that contain fructose are synthesized by family 32 (GH32) enzymes. However, the mechanisms whereby these enzymes diversify are not well understood. Here, we used a comparative genomics approach to investigate the evolution of GH70 and GH32 enzymes in the *A. kunkeei* population.

**Results:**

Based on phylogenetic inferences, the GH70 proteins in 38 reference *A. kunkeei* strains were sorted into glucan-binding enzymes, which were predicted to have glucansucrase and branching sucrase activities, and non-glucan binding enzymes of unknown enzymatic functions. Genes for the glucan sucrases and the branching sucrases are clustered in a chromosomal segment that also contains genes for GH32 enzymes. The number and combination of genes for the glucan-binding GH70 enzymes were mostly strain-specific, indicative of high rates of gene turnover. Neighboring genes often displayed a dramatic variability in synonymous and nonsynonymous substitution frequencies and have only rarely co-diverged. We identified short recombination tracts and a few long tracts that spanned across the cluster of genes for GH70 and GH32 enzymes. Genes encoding GH70 and GH32 enzymes evolve faster than genes encoding core proteins. The ratios of the relative effect of recombination to mutation for the core genome were estimated to 1.6 to 5.2 for *A. kunkeei* strains assigned to phylogroups A and B-C, respectively.

**Conclusions:**

Our results suggest genes for GH32 and GH70 proteins have a unique evolutionary history in each *A. kunkeei* strain and have diverged by duplications, deletions, fusions, recombination events and nucleotide substitutions. We suggest that genes for GH70 enzymes have escaped the homogenizing effects of homologous recombination to a greater extent than the core genes due to rampant gene gain and loss. The results imply that the clustering of the *A. kunkeei-*related strains into phylogroups mostly reflects the impact of homologous recombination on the core genome.

**Supplementary Information:**

The online version contains supplementary material available at 10.1186/s12866-025-04680-3.

## Background

*Apilactobacillus kunkeei* is a fructophilic lactic acid bacterium, which is abundant in the first segment of the honeybee gut that stores the nectar (the honey crop), as well as in the honeybee food sources and food products (honey, beebread, flowers and fruits) [1, 2]. The nectar is rich in fructose, glucose and sucrose, and some *A. kunkeei* strains can produce exopolysaccharides in the presence of sucrose, such as dextran [3]. The exopolysaccharides may provide a protective surface layer or promote cell adhesion and biofilm formation, but are not used as a source of nutrients [4]. Polysaccharides consisting of glucose moieties are synthesized by enzymes that belong to the glycoside hydrolase family 70 (GH70), whereas enzymes involved in the synthesis of fructose polymers belong to the glycoside hydrolase family 32 (GH32) [5, 6]. The aim of this study is to learn more about the mechanisms and evolutionary forces that generate diversity of the enzymes in the GH70 and GH32 families within *A. kunkeei*.

The structures and functions of enzymes in the GH70 family are well characterized due to the importance of exopolysaccharides for industrial applications. The most common types of GH70 enzymes are glucansucrases (GS), which catalyze the hydrolysis of sucrose to glucose and fructose, and then transfer the glucose moiety to a growing glucan chain such as dextran or to a non-carbohydrate substrate [7]. Branching sucrases (BrS) represent another subfamily of GH70 proteins that hydrolyze sucrose and add a variable proportion of α−1,2 or α−1,3-linked glucosyl branches onto dextran, but these enzymes are unable to synthesize α-glucans directly from sucrose in the absence of dextran [7, 8]. The GH70 proteins are about 1600 amino acids long and may either be single domain proteins or “twin” enzymes that contain both a GS and a BrS catalytic domain. All enzymes contain four core domains (I-IV) and the majority also contains auxiliary domains at either or both termini [9]. The auxiliary domain V is thought to play a role in α-glucan binding and has therefore been named the glucan-binding domain (GBD) [10–14]. The levels and activities of GSs are indicative of constitutive expression [11, 15, 16], testifying to their importance.

The GS and BrS enzymes are prevalent in lactic acid bacteria such as *Lactobacillus, Leuconostoc, Oenococcus, Weissela*, *Apilactobacillus, Fructobacillus* and *Nicoliella* [9]. A recent phylogenetic study based on protein alignments of the four core domains showed that the single BrS subclade was well separated from the many GS subclades [9]. This suggests that the diversity of these enzymes correlates with the reaction specificities of the enzymes rather than with the diversification patterns of the species. However, the mechanisms whereby enzymatic diversity of GH70 and GH32 family enzymes is generated within a species are not known.

*A. kunkeei* is an excellent model system to explore the within-species diversity of the GH70 and GH32 domain proteins. First, members of these protein families were found to be highly abundant in the supernatant fractions of *A. kunkeei* during cultivation on fructose-rich media [17]. Secondly, genome sequence information is available for more than 100 isolates obtained in pure culture [18–20]. Analyses of the exopolysaccharides in *A. kunkeei* strains H3 and H25 have shown that they are composed of dextran with 35% and 62% α−1,6 linked glucosyl units, respectively, which suggests the presence of branched structures [3]. Biochemical studies have shown that the branching sucrase BRS-D in *A. kunkeei* strain EFB6 catalyzes the formation of α−1,2 branched glucans in the presence of dextran [21, 22], whereas *A. kunkeei* strain DSM 12361 contains a twin enzyme, in which the second GH70 domain (GtfZ-CD2) catalyzes the formation of α−1,3-linked glucosyl units onto dextran [21]. Multiple genes for GH70 and GH32 enzymes are co-located in a segment of the *A. kunkeei* genome that was found to be excised from strain H3B2-03M during cultivation [18]. This suggests that the region encoding enzymes for exopolysaccharide synthesis in *A. kunkeei* is highly dynamic.

In this study, we have characterized the diversity of GH70 and GH32 domain proteins to learn more about mechanisms whereby these proteins evolve in the *A. kunkeei* population. The findings are discussed within the broader context of genome sequence divergence in *A. kunkeei*.

## Methods

### Sequence retrieval and prediction of domains and motifs

The *A. kunkeei* genome sequences were downloaded from NCBI on July 5, 2023 [18, 20, 23]. The genes coding for GH70 domain proteins were retrieved based on the locus tags associated to the GH70 domain family in Pfam annotations from previous InterProScan predictions as described in Dyrhage et al. [18], or from InterProScan v 5.59–91 predictions for strains DSMZ 12361 and IBH001, along with the positions of annotated Pfam domains. The same procedure was followed to retrieve the sequences of genes for GH32 proteins, although GH32 domain predictions were based on SMART annotations and the *scrB* gene was excluded due to its low similarity to the rest. The complete protein sequences were screened for known sequence motifs, whose position was confirmed by checking consistent placing in the alignment and the sequence similarity to previously described GH70 and GH32 proteins [8, 24]. When two domains were predicted in a protein encoded by single gene, the domains were numbered accordingly (i.e., H3B104J_13020_CD1 and CD2). SignalP6 [25] was run in its slow mode to retrieve signal peptide information. Nucleotide domain sequences were aligned to the sequences of the same family using EMBOSS Needle [26] to calculate pairwise percentages of identity.

To predict the positions of the glucan-binding domains, AlphaFold2.3.2 was run (with options -t 2025–05–14 -m monomer_ptm) [27] on a subset of the representative GH70 and GH32 protein sequences. Sequences were divided into shorter fragments with about 60 residues overlap. The residues in the output PDB files were renumbered and predicted aligned error (PAE) plots were created with scripts [28, 29]. The top ranked models were selected and visualized in UCSF ChimeraX 1.7.1 [30].

To check for possible sequencing errors, re-sequencing was done with PCR using the Phusion Hot Start II DNA Polymerase kit (F549) and dNTPs (R0192) (Thermofisher Scientific). Primer pairs were designed using Primer3Plus [31]. The samples were analyzed using a BIO-RAD T100 thermal cycler with a temperature gradient. PCR products were separated by 1% agarose gel electrophoresis, visualized with an AlphaImagerMini system and sequenced using the Mix2Seq kit (Eurofins Genomics).

### Alignments and phylogenetic analyses

The identification and clustering of core proteins were done as described in Dyrhage et al. [18], although replacing the assembly of *A. kunkeei* strain DSM 12361 to ASM1957599v1 and adding the assembly of *A. kunkeei* strain IBH001. The recombination filter was not applied to the dataset so as not to obscure the downstream analyses and interpretations of the observed sequence divergences. Protein sequences were aligned using MAFFT v7.475 [32], forcing the L-INS-i strategy, and the core protein trees were generated using the iqtree2 command from IQtree multicore v2.1.4-beta [33], with the “-msub nuclear” option and ModelFinder [34], whereas the glycosyl hydrolase trees were generated using the iqtree command with the same options. The WAG + F + I + G4 model was used for the GH70 tree and the Q.pfam + F + G4 model for the GH32 tree. For the trees inferred based on the GH70 domain subtypes, LG + F + R2 was used for the GS1 tree, LG + F + G4 for the GS2 tree, WAG + F + G4 for the BrS tree and JTT + F + I for the NGB tree. In the GH70 tree, GtfB-like domains from related species (*Limosilactobacillus reuteri* and *Limosilactobacillus fermentum*) taken from Meng et al. [21] were retrieved from InterProScan v5.59 annotations and used to root the tree, whereas the GH32 phylogeny was rooted on its longest branch. Branch supports were assessed with 1000 ultrafast bootstrap replicates with a hill-climbing nearest neighbor interchange search [35]. Figures were drawn with Ete3 [36].

For comparison of tree topologies, the single protein domain phylogenies were midpoint rooted and compared to the core protein trees with Dendroscope, version 3.8.4 [37], using default parameters. QuartetScores version 1.0.0 was used for quantitative comparisons of the tree topologies [38]. The unrooted core protein phylogenies were used as reference trees and the GS1 and NGB domain phylogenies were used as evaluation trees, respectively. The results files were re-formatted using a script obtained from the QuartetScores Github [39] and displayed and midpoint rooted in Dendroscope version 3.8.4. For comparisons of gene order structures, the sequences of the genomic regions were aligned with BLASTn [40], following the order of the core protein phylogeny. The synteny comparisons were visualized with PyGenomeViz v1.5.0 [41]. In four strains, a segment containing phage genes was excluded from the genomic regions that contained a single gene for a GH70 protein.

### Searches for homologs to GH70 domain proteins in other species

Searches for homologs to the GH70 domain proteins were carried out against the RefSeq Protein database using BLASTp, and limiting the search to *Bacillus, Lactobacillus* and *Streptoccocus* and excluding hits to *A. kunkeei* and unknown genera. The GS2 protein domain from H1B3-02M (H1B302M_12900) was used as query, with an E-value threshold of 10^–5^, a percentage of identity ≥ 41%, to exclude GtfB-like or more divergent proteins, and query coverage of ≥ 95%, to exclude hits with incomplete GH70 domains. BLASTp searches using the GS1, BrS and NGB proteins from strain H1B3-02 M as queries (H1B302M_12880, H1B302M_12890 and H1B302M_04960, respectively) were also carried out as described above. 3235 sequences were retrieved from the search with the GS2 protein as the query and an additional 18, 2 and 1 proteins were added, respectively, from the other searches.

The retrieved sequences were sorted by species, percentage of identity to the query and accession number, in this order. The species with the most hits was chosen as representative for each genus, except in *Apilactobacillus*, from which all species were used as representatives. For *Weissella*, *W. confusa* was selected as representative because of potential sequencing errors in some of the *W. cibaria* sequences. Hits to uncultured species were excluded. If several species had the same number of hits, the species with the widest range of percentages of identity with the query was chosen. In cases of one hit per species in a genus, the hit with the highest percentage of identity was chosen. If there was no distinction in the number of hits or in the percentage identity, the species was chosen in alphabetical order. From the selected species, matches were retrieved in intervals of 2% decreases in the percentage of identity with the query. One hit from *Leuconostoc citreum* and two from *Streptococcus* were added from the GS1 and BrS/NGB searches, respectively. Sequences of known function were taken from Meng et al. [21] and added to the BLASTp dataset, choosing one sequence per protein function and representative species in the BLASTp dataset. An experimentally confirmed α−1,2 BrS from *A. kunkeei* (NCBI ID: KDB00248.1) [22] was also added to the dataset. The GH70 domains of the newly-retrieved sequences were recovered based on InterProScan v 5.59–91 annotations of the complete protein sequences. The domains served as inputs to retrieve motif sequences and generate a tree (Q.pfam + F + R6) as described for the *A. kunkeei* GH70 proteins.

### Measures of nucleotide substitution frequencies

Alignments of the core proteins and the subtypes of GH70 protein domains found in ≥ 10 strains were converted into DNA alignments without gaps using PAL2NAL v14 [42], with -output paml and nogap options. The pairwise substitution frequencies were computed on the PAL2NAL output alignments using CodeML from the PAML v4.9 subpackage [43, 44], with options CodonFreq = 2 (F3 × 4 model), model = 1 (one omega ratio per branch), ncatG = 8 (number of gamma categories) and an initial omega and kappa of 0.7 and 2, respectively. CodeML was run within Biopython v1.79 [45] in Python v3.8.6 [46]. General statistics (mean and median *d*_*N*_, *d*_*S*_ and *d*_*N*_*/d*_*S*_) were calculated for each type of GH70 and GH32 domain. For core genes, average statistics between each pair of strains were calculated. In all cases, CodeML was run in the same isolated Anaconda v1.9.0 environment [47]. For the four most common GH70 subtypes, the pairwise *d*_*S*_ and *d*_*N*_ values were compared against each other by generating scatterplots with Matplotlib v3.9.2 [48]. Pairwise *d*_*S*_ values > 1.5 were set to 1.5. Linear regressions and Pearson correlation coefficients were calculated with numpy.polyfit and scipy.stats.pearsonr, respectively, for each comparison where *d*_*S*_ < 1.5. The distributions of pairwise *d*_*S*_ values of the gene subtypes were also plotted as histograms over the distribution of pairwise *d*_*S*_ values of core genes. The same set of strain pairs was used for the core gene and gene subtype distributions. For each comparison, the two-sample Kolmogorov–Smirnov test was performed with the stats.ks_2samp function to test if the distribution of pairwise *d*_*S*_ values within gene subtypes was statistically different from the pairwise *d*_*S*_ distribution of core genes.

For comparisons of SNPs in the ~ 25 kb genomic segment, the amino acid sequences of the encoded proteins were aligned with MAFFT v7.475 L-INS-i and converted into DNA alignments using PAL2NAL v14, with the -output fasta option. The alignment was visualized by comparing the translation of each codon position, using Matplotlib v3.3.4 and the heatmap function from Seaborn v0.13.2 [49].

### Recombination analysis

The nucleotide sequence of the segment of the genome from the gene upstream of *mntH* to *cds5*, the CDS upstream of *S3*, was retrieved and aligned using MAFFT v7.475 L-INS-i. The same was done for the segment between *cds7*, upstream of *nox*, and *bcrA*. The two alignments were merged, indicating the position where they were joined, and loaded into RDP5 v5.58 [50]. RDP5 was run using default options, but indicating that the input sequences were linear and using all the recombination detection methods except for LARD [50–57]. Bootscan and Siscan were set to both predict recombination events and to test the events predicted by other methods. Events shorter than 1000 nucleotides were discarded unless they overlapped with the start or end positions of one of the alignments.

The 38 *A. kunkeei* genome files were used as input sequences for the ClonalFrameML v1.13 analysis [58]. For the MP2 strain, the reverse complements were used, and ORI was predicted using Circlator v1.5.5 (option “fixstart”) [59], with default settings. The genome files for the phylogroup A and B-C strains were aligned, respectively, using progressiveMauve (version 2015–02–13) and stripSubsetLCB (option “500”) to filter out everything but > 500 bp blocks present in all genomes [60]. The strain phylogeny was pruned to create phylogenies of the phylogroup A and B-C strains, respectively, using iTOL v7 [61]. The filtered genome alignments and the corresponding phylogenies were then analyzed using ClonalFrameML (options “-emsim 100 -xmfa_file true”).

## Results

### Gene datasets for GH70 and GH32 proteins

As the starting dataset, we used the genomes of 106 *A. kunkeei* isolates (Supplemental Table S1). This dataset contained a total of 266 genes that code for proteins with 272 GH70 domains and 69 genes that code for proteins with GH32 domains, according to InterProScan annotations. For more detailed comparative analyses, we used a smaller set of 38 non-redundant *A. kunkeei* strains (Supplemental Table S2). This set included most of the 34 *A. kunkeei* strains shown previously to represent the genetic diversity of the full set of 106 strains [18], to which we added two strains from the same strain collection which contained novel variants of the GH32 proteins [18] and another two strains sampled elsewhere, DSMZ 12361 [23], which contained GH70 proteins with experimentally determined enzymatic activities [21], and IBH001.

The 38 reference genomes encoded 92 proteins with full-length GH70 domains of 764 to 811 amino acids, 87 of which contained a single GH70 domain and the remaining 5 proteins contained two GH70 domains, and an additional 25 proteins with a single GH32 domain (Supplemental Tables S3, S4). The total sizes of the GH70 enzymes ranged from 1021 to 2206 amino acids for proteins with a single full-length GH70 domain, and from 2865 to 2984 amino acids for proteins with two GH70 domains (Supplemental Table S3). Of the 92 proteins in total, as many as 65 contained auxiliary glucan-binding repeats (PF19127). The remaining 27 enzymes without glucan-binding repeats are here referred to as the non-glucan-binding (NGB) proteins. Five strains contained genes for GH70 enzymes with internal termination codons that were confirmed by resequencing (Supplemental Fig. S1, Table S5). Of the 25 proteins with a single GH32 domain, 17 contained glucan-binding domains (Supplemental Table S4). The GH32 enzymes ranged from 942 to 1761 amino acids in length, the longest of which contained 11–12 repeats of around 44 amino acids at their C-terminal ends. The *A. kunkeei* genomes also contained a conserved gene for a protein about ~ 90 amino acids that showed similarity to the N-terminal ends of the GH70 domains in the other proteins, but which was not included in the comparative analyses below.

### Diversification patterns of the GH70 and GH32 domains

**Fig. 1:**
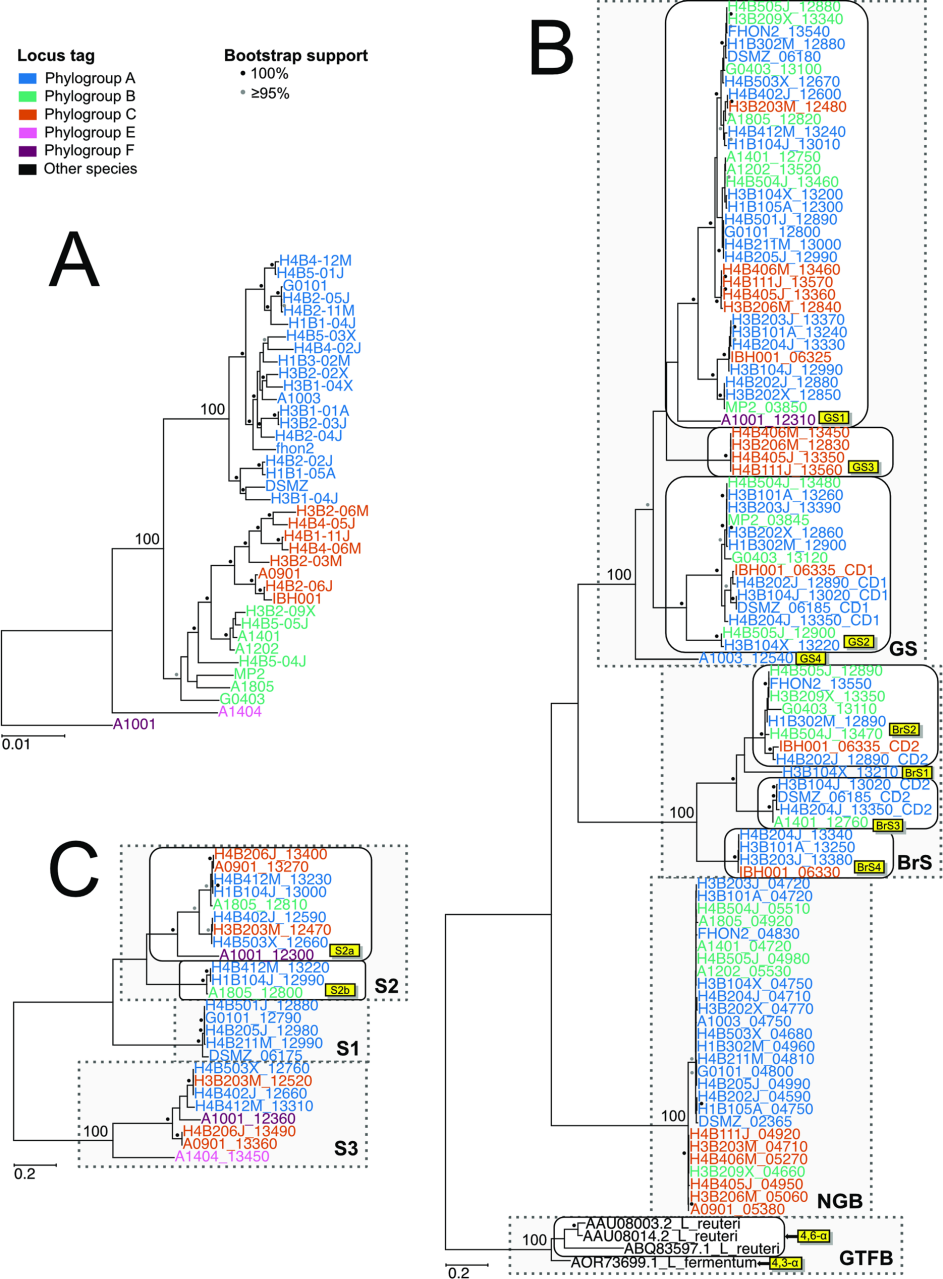
Phylogenetic relationships of GH70 and GH32 domains. Phylogenetic trees were inferred from the amino acid alignments of the **A**) 907 core proteins, **B**) 97 GH70 domains plus four outgroups and **C**) 25 GH32 domains in 38 representative strains of *A. kunkeei*. Strain names and locus tags are colored by phylogroup designations according to the core protein phylogeny by Dyrhage et al. [18]. Proteins annotated as GTFB in *Limosilactobacillus reuteri* and *Limosilactobacillus fermentum* were used as outgroups in the GH70 domain phylogeny. The trees were inferred with maximum likelihood methods. Bootstrap support values of 100 and ≥ 95 are shown as black and grey dots, respectively. The phylogenies with exact bootstrap support values ≥ 50 are shown in Supplemental Fig. S2

Maximum likelihood phylogenies based on the amino acid sequences of the GH70 and GH32 domains, respectively, showed that enzymes with and without glucan-binding repeats diverged into two distinct clades (Fig. 1; Supplemental Fig. S2). For comparison, a phylogeny was also inferred based on the concatenated alignment of 907 core proteins shared by all 38 strains, which showed that 36 strains belonged to phylogroups A, B and C, while phylogroups E and F were represented by a single strain each (Fig. 1A; Supplemental Fig. S2A). The tree topology of the 97 GH70 domains showed two distinct clades, one of which contained the 70 domains extracted from proteins with glucan-binding repeats, while the other clade contained the 27 domains derived from proteins without glucan-binding repeats (Fig. 1B; Supplemental Fig. S2B). Likewise, the tree topology of the 25 identified GH32 domains revealed two main clades, one of which contained the domains extracted from proteins with glucan-binding domains, while the other clade was composed of the domains associated with proteins that lacked glucan-binding domains (Fig. 1C; Supplemental Fig. S2C).

The clade consisting of GH70 domains derived from enzymes with glucan-binding domains comprised two subclades with predicted different catalytic activities (Fig. 1B; Supplemental Figs. S2B, S3A). One subclade, which was composed of 53 domains, contained the catalytic domain CD1 of GtfZ in strain DSMZ 12361 and the domains in this clade were inferred to have glucan sucrase (GS) activities. The other subclade, which was composed of 17 domains, contained the catalytic domain CD2 of GtfZ in strain DSMZ 12361, which has been associated with branching sucrase activity (BrS) and the domains in this clade were therefore inferred to have branching sucrase activities. The GH70 domains derived from proteins with glucan-binding domains were further divided into several subtypes (GS1-GS4, BrS1-4) based on highly supported smaller clades in the phylogeny. The clade consisting of the GH70 domains extracted from enzymes without glucan-binding domains included no sequences with experimentally known functions.

All complete GH70 domains contained the seven characteristic sequence motifs of previously described GS and BrS proteins from *Lactobacillaceae* species [6, 8], including the amino acids that represent the catalytic triad (Supplemental Table S6). A comparison of the amino acid sequences in the motifs II, III and IV in the GS and BrS domains, respectively, confirmed their suggested enzymatic functions (Table 1). Furthermore, the sequence motifs in the BrS2 domains were identical to the α−1,2 BrS domain in the BRS-D protein of EFB6, and the sequence motifs in the BrS3 domain were identical to the α−1,3 BrS domain in the GtfZ-CD2 protein of DSMZ 12361. The sequence motifs in the GH70 domains of the NGB proteins were similar but not identical to the motifs in either of the GS and BrS domains. Likewise, the GH32 domains derived from proteins with glucan-binding domains were divided into subtypes based on highly supported smaller clades in the phylogeny (S1, S2), whereas the GH32 domains derived from proteins that lacked glucan-binding repeats were named the S3 subtype (Fig. 1C; Supplemental Figs. S2C, S3B). The eight characteristic sequence motifs were identified in all GH32 domains and the three subtypes contained similar but not identical motifs (Supplemental Table S7).

**Table 1:** Motif comparison with previously described sequences

Protein	Motif I	Motif II	Motif III	Motif IV	Motif V	Motif VI	Motif VII
GS	XDFVPDQ	XRX**D**AXDNXX	HXXIL**E**DWSXND	IXRAH**D**XGVQXXI	EFLLXXDVDNSNP	LGXTXXELXPQY	TTPRXYYGD
GtfZ-CD1	ADIVPDQ	VRV**D**AVDNMD	HLSIL**E**DWSGND	IVRAH**D**AGVQDII	EFLLANDVDNSNP	LGFTYLELPPQY	TTPRVYYGD
BrS2	ADVVYNQ	IRI**D**AVDFIS	HISLV**E**GGVDAG	IVHAH**D**KDVQEKV	DFLLANDVDNSNP	WGVTSFEMAPQY	TVPRIYYGD
BRS-D	ADVVYNQ	IRI**D**AVDFIS	HISLV**E**GGVDAG	IVHAH**D**KDVQEKV	DFLLANDVDNSNP	WGVTSFEMAPQY	TVPRIYYGD
BrS3	ADVVYNQ	IRI**D**AYAFIN	HLSIV**E**AGVDAG	IVHAH**D**KDIQDKV	DFLLANDVDNSNP	WGITSFELPPQY	TVPRIYYGD
GtfZ-CD2	ADVVYNQ	IRI**D**AYAFIN	HLSIV**E**AGVDAG	IVHAH**D**KDIQDKV	DFLLANDVDNSNP	WGITSFELPPQY	TVPRIYYGD
BrS & GS	XDXVXXQ	XRX**D**AXXXXX	HXXIX**E**XXXXXX	XXXAH**D**XXXQXXX	XDLLXXDVDNSNP	XGXTXXEXXPQY	TXPRXXYGD
NGB	TDYVMNQ	GRE**D**ATGSMD	HLNLL**E**DGAPES	FLNVH**D**-GVQTFI	ELLLANDIDNSNP	LGITDFEFPGHY	VVPRVYYGD

Comparison of consensus motifs from subtypes GS, BrS2 and BrS3 to previously described sequences. The NGB motifs are compared to the GS and BrS consensus

### Phyletic distribution patterns of genes for GH70 and GH32 proteins

**Fig. 2:**
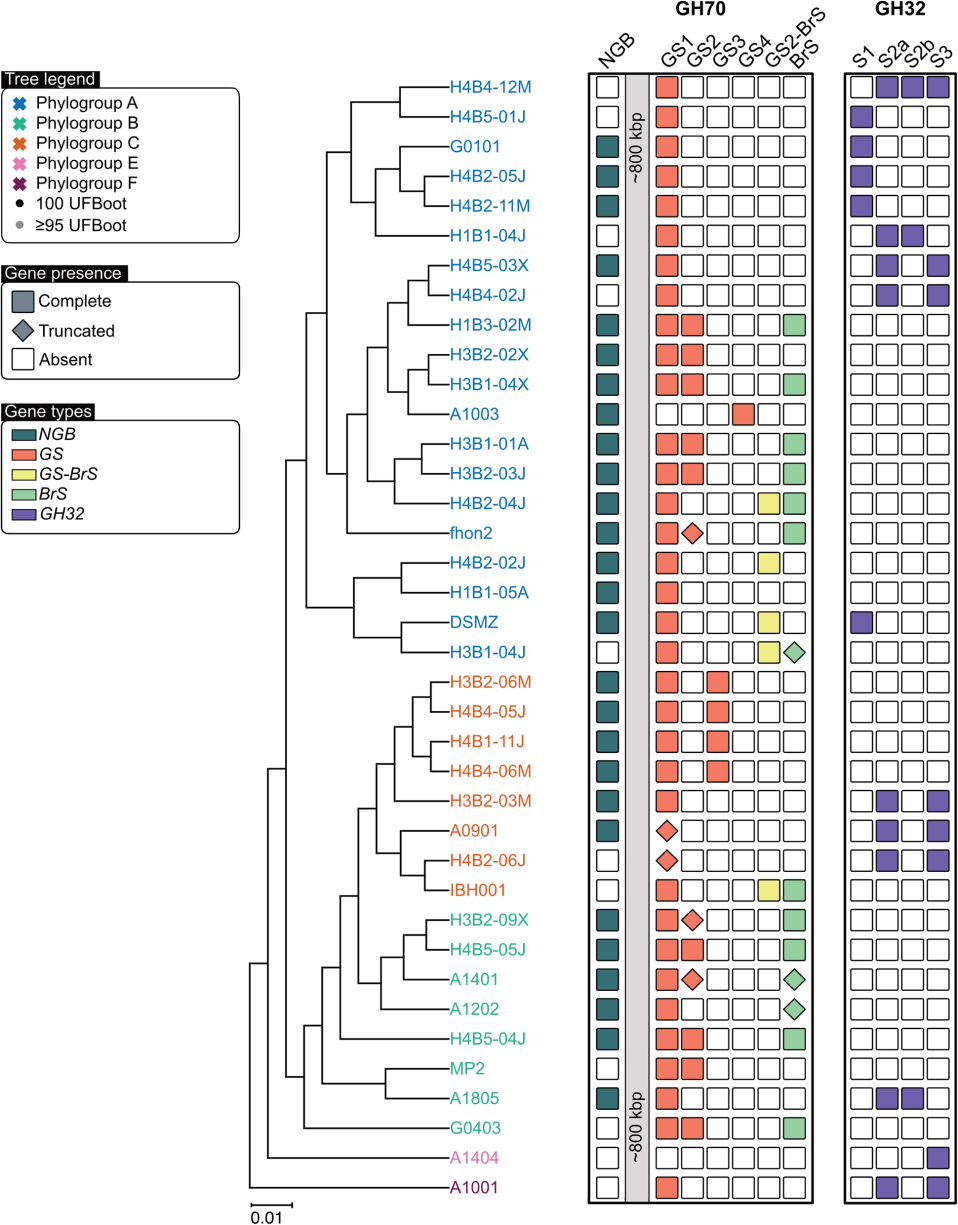
Phyletic distribution pattern of genes for GH70 and GH32 family proteins. The presence/absence patterns of genes for GH70 and GH32 family proteins mapped onto the core protein phylogeny of representative strains of *A. kunkeei* shown in Fig. 1A. Strain names are colored by phylogroup designations. Complete genes are shown as colored squares, truncated and degraded genes as diamonds and absent genes as blank squares. GS = glucansucrases, BrS = branching sucrases, NGB = non-glucan binding GH70, S1-3 = GH32 subtypes, of which S3 lacks glucan-binding domains

We mapped the content of genes for the different types of GH70 and GH32 enzymes onto the core genome phylogeny of the *A. kunkeei* strains (Fig. 2). Genes for the NGB and GS1 proteins were widely distributed, whereas the other genes for GH70 and GH32 proteins showed patchy distribution profiles. This set of genes included seven putative pseudogenes which contained short N-terminal fragments or mutations that disrupted the open reading frame. Strain A1404 was the only strain in our dataset that contained no genes or gene fragments for GH70 enzymes.

The genes for GH70 and GH32 proteins with glucan-binding domains formed a conserved cluster of genes with the order “GS2_BrS_GS1_GS3/S1/S2a_S2b” (Fig. 4; Supplemental Fig. S4). The gene for the non-glucan-binding GH32 enzyme S3 was located shortly upstream of this gene cluster, whereas the gene for the NGB protein was located elsewhere (Fig. 4A; Supplemental Fig. S5). Interestingly, the BrS and GS2 domains mostly co-occurred (Figs. 4CD; Supplemental Figure S4). In 11 genomes, the gene encoding the GS2 domain was located immediately upstream of the gene encoding the BrS enzyme, and in another 5 genomes the two domains were fused such that they belonged to the same open reading frame. Three genomes that encoded a twin protein also encoded an additional protein with a single BrS domain. Strain A1401 contained only the N-terminal end of the gene for GS2 and a mutated gene for BrS, indicating that both genes are in the process of deterioration. It was also notable that both the GS2 and the GS2-BrS proteins contained serine-rich repeats of about 15 amino acids at their C-terminal ends (Supplemental Fig. S3A).

**Fig. 3:**
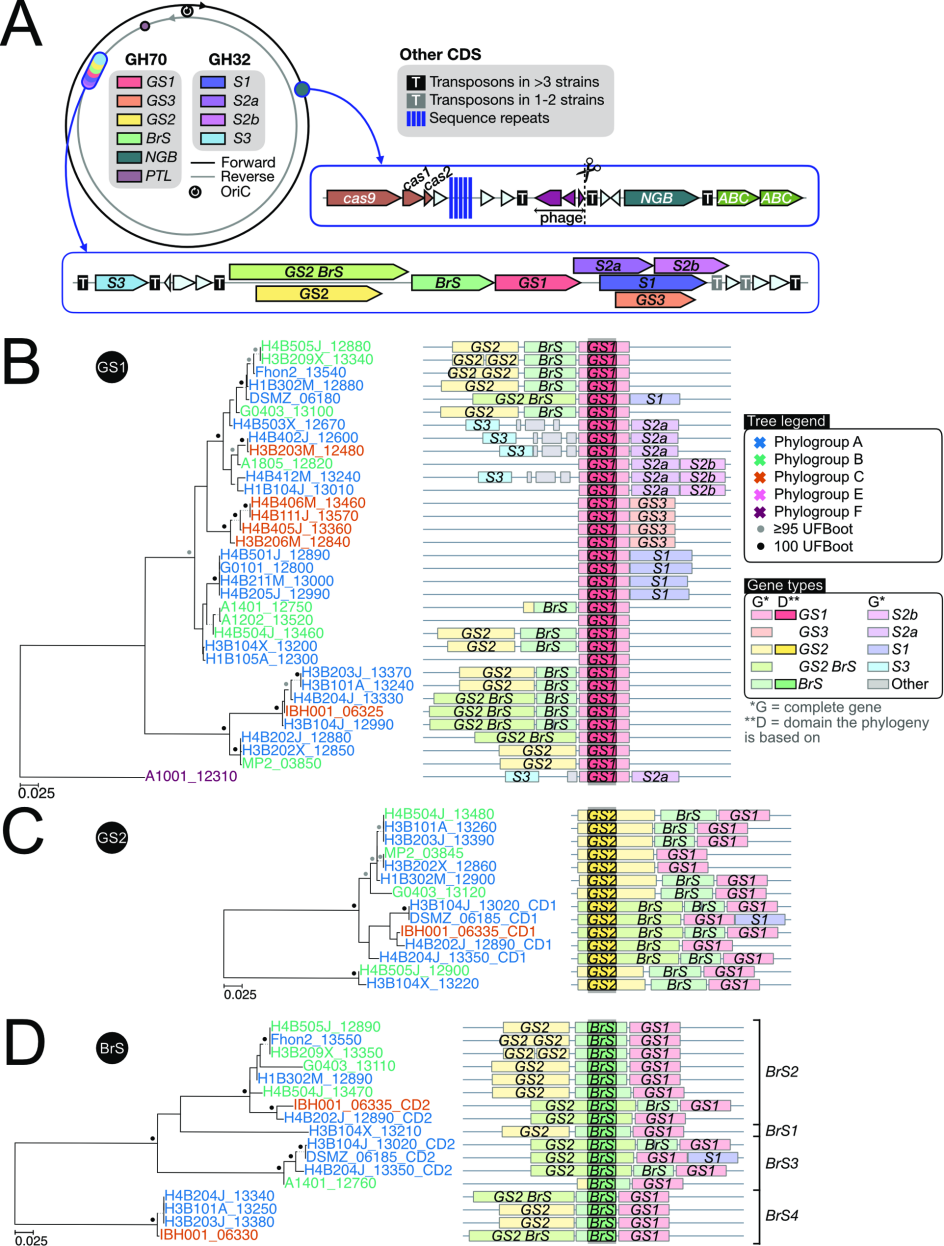
Schematic illustration of the order of genes for GH70 and GH32 family proteins. The location of genes for GH70 and GH32 enzymes have been mapped onto **A**) a circular representation of the *A.* *kunkeei* genome, detailing the order and direction of genes and transposons in the segment containing the majority of genes for GH70 and GH32 proteins and the segment containing the NGB gene, where other genes of interest are also highlighted, such as phage genes, cas endonucleases and genes for transport systems, and** B-D**) the GH70 domain phylogenies. The order of genes is shown for genomic segments that contain genes coding for the **B**) GS1, C) GS2 and **D**) BrS domains. Locus tags are colored by phylogroup designations. Bootstrap support values of 100% are shown as black dots, and values ≥ 95% are shown as grey dots. GS = glucansucrases, BrS = branching sucrases, NGB = non-glucan binding GH70, PTL = proteins with the N-terminal segment of a GH70 domain, S1-3 = GH32 subtypes, of which S3 lacks glucan-binding domains

### Origin and divergence of genes for GH70 enzymes

**Fig. 4:**
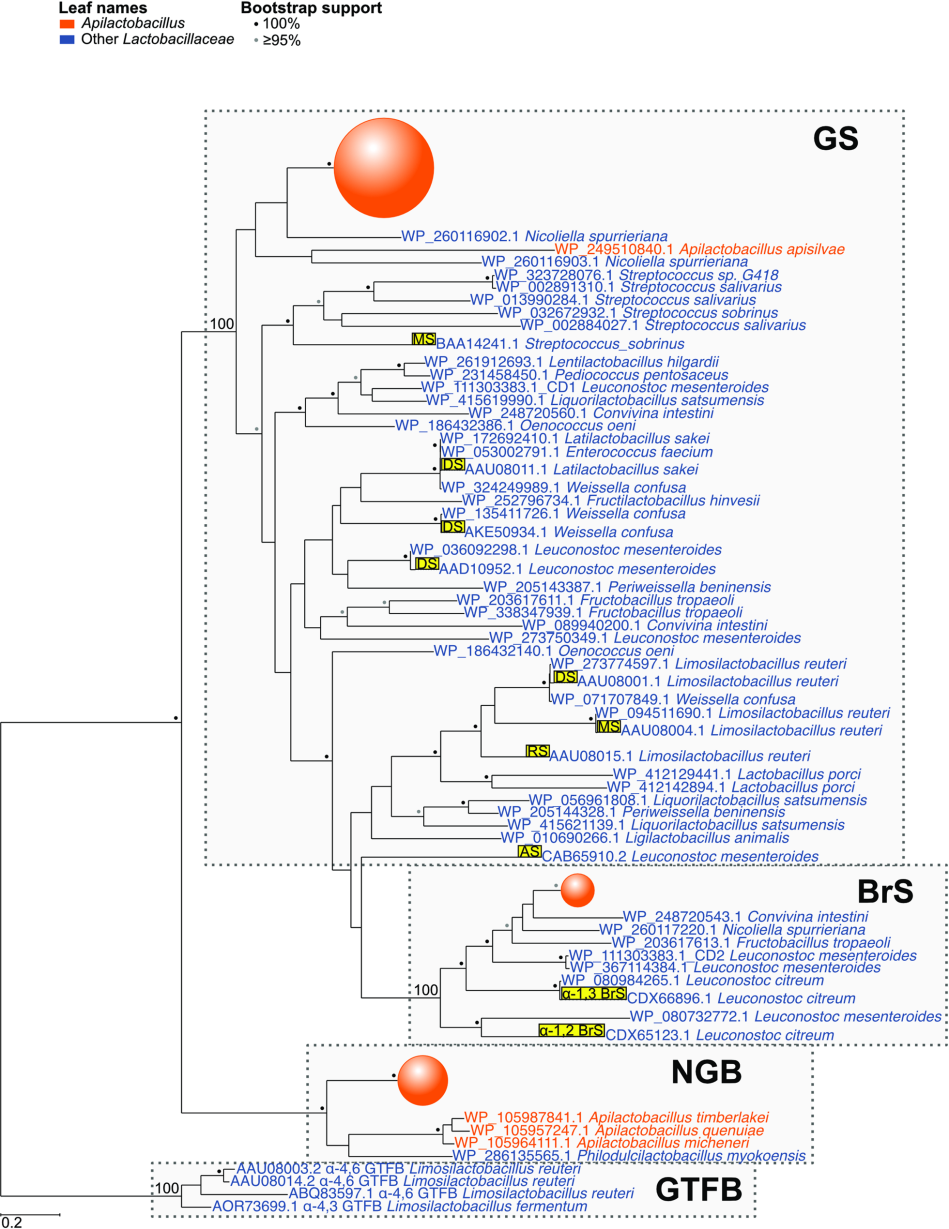
Phylogenetic relationships of GH70 domains in bacteria. Phylogenetic trees were inferred with the maximum likelihood method from the amino acid alignments of GH70 domains in members of the *Lactobacillaceae, Streptococcaceae* and *Enterococcaceae*. The clades containing GSs, BrSs and NGBs in the reference *A. kunkeei* strains and *A. apinorum* are schematically indicated by circles colored in orange. The catalytic activities of protein domains with experimentally confirmed functions are indicated by AS (alternansucrase), DS (dextransucrase), MS (mutansucrase), RS (reuteransucrase) and BrS labels, marked in yellow. Proteins annotated as GTFB in *Limosilactobacillus reuteri* and *Limosilactobacillus fermentum* were used as outgroups. The domain subtypes (GS, BrS, NGB or GTFB) are indicated by dotted rectangles. Bootstrap support values of 100% and ≥ 95% are shown as black and grey dots, respectively. The phylogenies with complete bootstrap support values ≥ 50 are shown in Supplemental Fig. S6

A phylogenetic tree inferred from the GH70 domains showed that the GS, BrS and NGB enzymes in *A. kunkeei* and *A. apinorum* formed three distinct monophyletic clades, each of which was a sister clade to GH70 enzymes in other bacterial species (Fig. 4; Supplemental Fig. S6; Table S8). The GS clade in *A. kunkeei* clustered with GH70 enzymes with confirmed glucansucrase activities in, for example, *Latilactobacillus sakei, Weisella confusa* and *Leuconostoc mesenteroides*, whereas the BrS clade in *A. kunkeei* clustered with GH70 enzymes with confirmed BrS activities in *Leuconostoc citreum*. The NGB clade in *A. kunkeei* represented also a distinct clade, which however only had homologs in *Apilactobacillus* species and *Philodulcilactobacillus myokoensis*, none of which had an experimentally confirmed function. Thus, despite the inclusion of homologs from other bacterial phyla, the GH70 enzymes with and without glucan-binding repeats were well separated from each other.

**Fig. 5:**
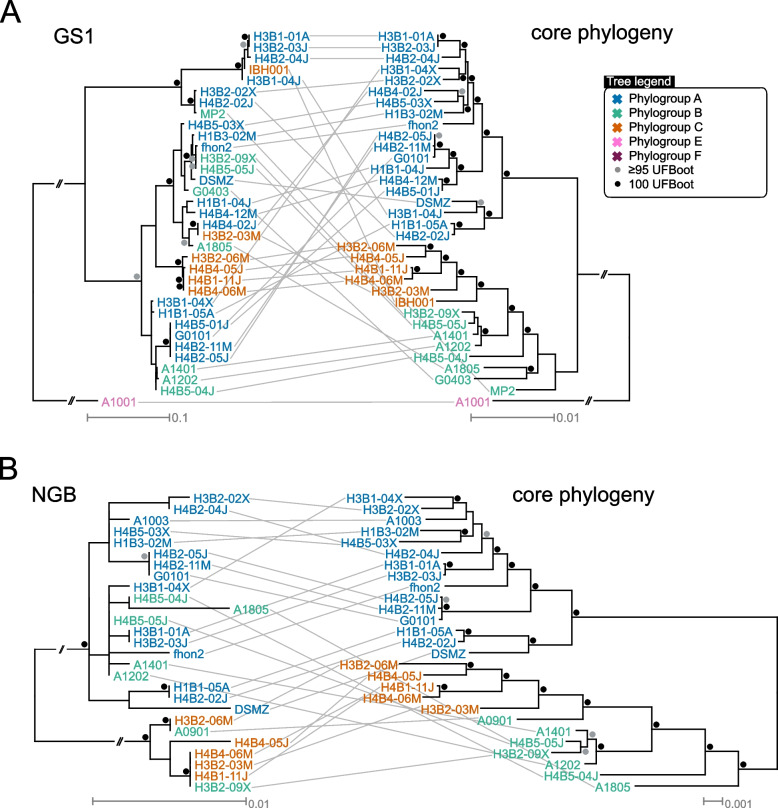
Comparison of GH70 domain tree topologies. Phylogenetic trees were inferred based on the amino acid alignments of the **A**) GS1 and **B**) NGB domains in the two subsets of *A. kunkeei* strains that contain genes for these proteins. The tree topologies were compared to the topologies obtained from phylogenetic inferences based on 907 core proteins in the two subsets of *A. kunkeei* strains, respectively. Strain names and locus tags are colored by phylogroup designations. The trees were inferred with the maximum likelihood method. The longest branches have been cut for ease of comparison. Bootstrap support values of 100% and ≥ 95% are shown as black and grey dots, respectively. The phylogenies with complete branch lengths are shown in Supplemental Fig. S7

A comparison of the tree topologies obtained from GS1 and NGB domains in *A. kunkeei* showed that they were incongruent with the protein tree inferred from 907 core proteins which are conserved in all *A. kunkeei* strains (Fig. 5; Supplemental Fig. S7). The total set of core proteins was used in this analysis, without filtering out putative recombinants so as not to inadvertently bias the results. The incongruences were visualized using so-called “tanglegrams” (Fig. 5; Supplemental Fig. S7AB) and quantified using quartet nodes and internode certainty scores (Supplemental Fig. S7CD). The same subset of strains was used in each pairwise comparison. Furthermore, although the genes for GS2 and BrS domains mostly co-occurred in the same genome, the trees provided no indications of co-diversification. For example, the GS2 domains in the GS2-BrS fusion proteins formed a distinct clade in the GS2 tree, whereas the BrS domains in the same proteins segregated into two different subclades in the BrS tree (Fig. 1B; Supplemental Fig. S2B). The BrS domains in the GS2-BrS fusion proteins have also not co-diverged with the BrS domains encoded by the downstream gene in the few genomes that encode both a twin and a single BrS domain protein.

**Fig. 6:**
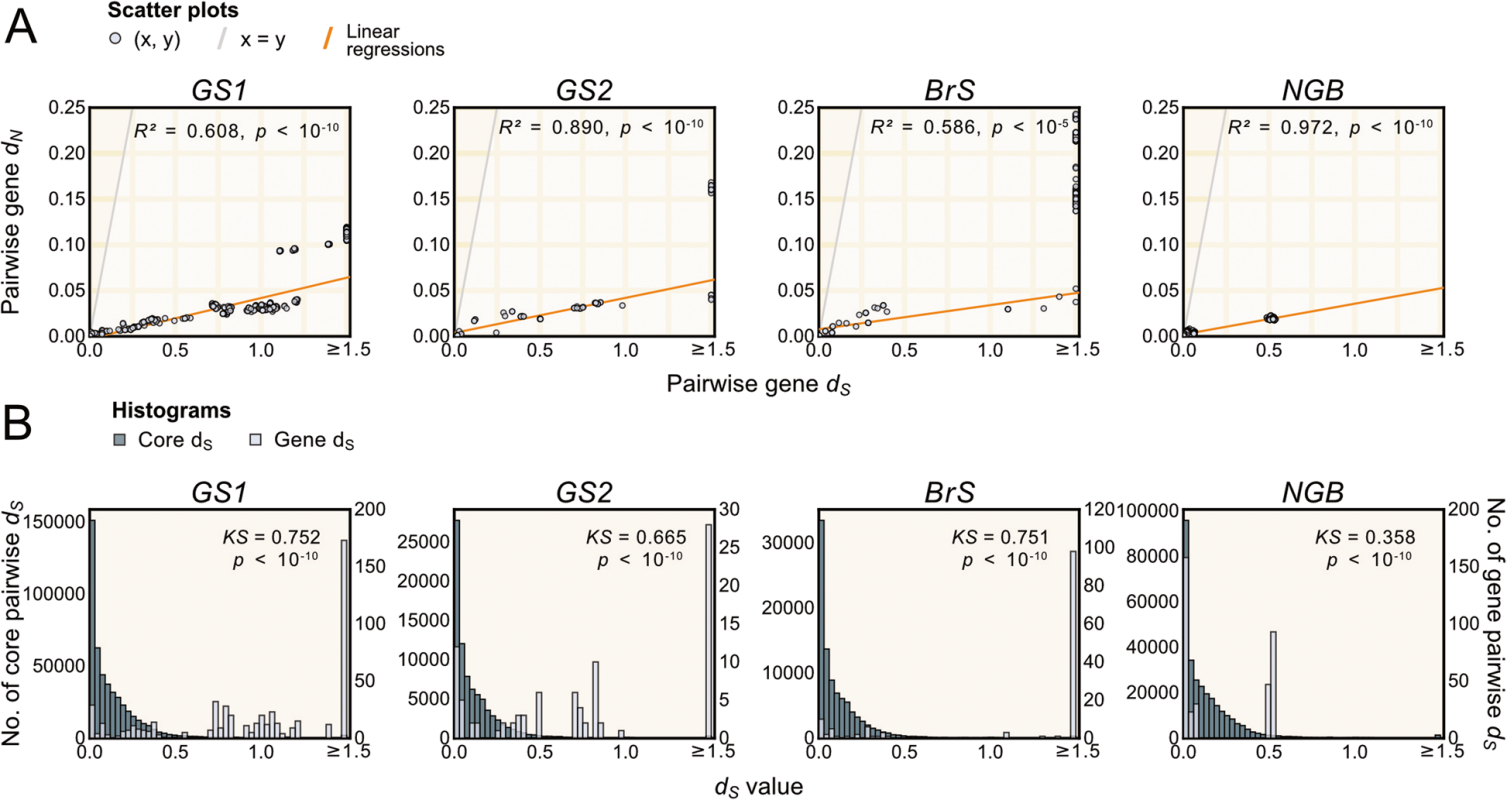
Substitution frequency estimates of gene sequences for GH70 domains. **A** The synonymous (*d*_*S*_) substitution frequencies of the gene sequences for the GS1, GS2, BrS and NGB domains were plotted against the nonsynonymous substitution frequencies (*d*_*N*_) of the same sequences. **B** The distribution profiles of the *d*_*S*_ values of the gene sequences for the GS1, GS2, BrS and NGB domains were compared to the distribution profiles of the 907 core genes. The same subset of strains was used for the pairwise comparisons in each column. The correlation coefficient (R^2^) or Kolmogorov-Smirnow statistic (KS) and the p-value for each comparison are shown within the plots

The non-synonymous (*d*_*N*_) substitution frequencies were on the average tenfold lower than the synonymous (*d*_*S*_) frequencies for the GH70 domain sequences, confirming that they evolve under strong purifying selection (Fig. 6A; Supplemental Table S9). However, the distribution profiles of the* d*_*N*_ and *d*_*S*_ values for the GH70 domain sequence were the opposite extremes of the distribution profiles of the core genes (Kolmogorov–Smirnov test; *p* < 10^–10^) (Fig. 6B). Whereas the majority of core genes were identical or nearly identical in the many pairwise sequence comparisons, the majority of sequences for the GS1, GS2 and BrS domains were saturated for substitutions at synonymous sites. The sequences encoding the NGB domain presented an interesting case in that the *d*_*S*_ values were either close to zero or equal to 0.5 substitutions per site, consistent with the phylogeny which showed that the GH70 domains segregated into two clades with near sequence identity between strains within each clade.

The *d*_*S*_ values of the individual GS1, GS2 and BrS domain sequences in pairs of genomes that encoded two or more domains were also very different, indicative of local variability in the sequence divergence patterns. For example, the substitution frequencies of the GS2 domain sequences (*d*_*N*_ = 0.018, *d*_*S*_ = 0.12) were tenfold lower than the substitution frequencies of the BrS domain sequences (*d*_*N*_ = 0.15, *d*_*S*_ ≥ 1.5) in strains H4B2-02 J and H3B1-04 J (Supplemental Table S9). Interestingly, it was predicted that the BrS domain in strain H4B2-02 J has an alpha-1,2 enzymatic activity, whereas the BrS domain in strain H3B1-04 J was predicted to have an alpha-1,3 enzymatic activity (Fig. 1B).

### Influence of recombination to the divergence of the GH70 genes

**Fig. 7:**
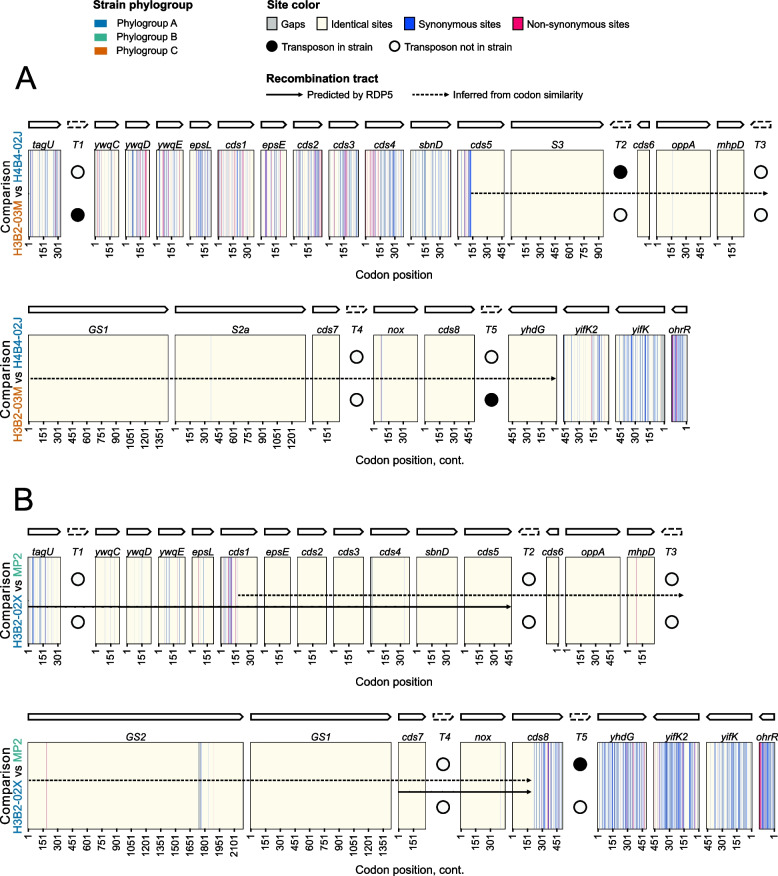
Examples of long recombination tracts in the genomic region encoding GH70 enzymes. The location of single nucleotide polymorphisms at synonymous (blue lines) and nonsynonymous (pink lines) sites in a segment of the *A. kunkeei* genome that includes genes for GH70 family proteins in comparisons of strains **A**) H3B2-03 M and H4B4-02 J and **B**) H3B2-02X and MP2. The location of transposons in some of the representative strains of *A. kunkeei* is indicated with open circles, and these are colored black in strains that contain a transposon at this site. The solid black arrow indicates the span of the recombination event as predicted by RDP5, and the dotted arrow indicates the span of the event inferred based on the codon alignment

A few long recombination tracts that extended across the region coding for glucan-binding GH70 and GH32 proteins were inferred. For example, a putative recombination tract of 24 kb was predicted that extended from the gene for *cds5* to the N-terminal end of the gene for *ydhG* in the phylogroup A strain H4B4-02J and the phylogroup C strain H3B2-03M (Fig. 7A). In total, only eight synonymous and three nonsynonymous substitutions were detected in the genes located within the segment. Likewise, a putative recombination tract of 26 kb that extended from the middle of the *cds1* to the middle of *cds8* was predicted in the comparison between the phylogroup A strain H3B2-02X and the phylogroup B-C strain MP2 (Fig. 7B). Five synonymous and five nonsynonymous substitutions were noted in the genes located within this segment, indicative of a recent recombination event. A systematic search for recombination events with the aid of RDP5 [50–57] confirmed the recombination tract between strains MP2 and H3B2-02X and provided support for a few recombination hotspots above the 95% confidence interval in this region, mostly at sites that contained transposons in some strains (Supplemental Fig. S8; Supplemental Table S10).

Overall, the impact of recombination to the divergences of the *A. kunkeei* genomes within phylogroup A and phylogroups B-C was estimated to be 1.6-fold to fivefold higher than the impact of single nucleotide substitutions (Supplemental Fig. S9; Supplemental Table S11). These estimates were based on the use of ClonalFrameML [58] and genome fragments of more than 500 nucleotides shared by all reference genomes within phylogroup A and phylogroup B-C, respectively. The sequence divergences of the *A. kunkeei* genomes (ν) were on the average estimated to 0.05–0.08 substitutions per site and the ratio of recombination relative to mutation rate (R/θ) was estimated to 0.15–0.20. Furthermore, the sizes of the recombination tracts (δ) were estimated to 136 nucleotides for the phylogroup A genomes and to 466 nucleotides for the phylogroup B-C genomes. Based on these results, the average number of substitutions introduced per recombination event (δν) were estimated to 10 for the phylogroup A genomes and to 26 for the phylogroup B-C genomes. Finally, the ratio of the relative effect of recombination and mutation to the sequence divergences (*r/m*) were estimated to 1.55 for phylogroup A genomes and to 5.23 for phylogroup B-C genomes (*r/m* = (R/θ) × δ × ν).

## Discussion

We have studied the mechanisms whereby the diversity of proteins with domains from glycoside hydrolase families 70 and 32 is generated, using *A. kunkeei* as our model species for which complete genome data from multiple strains are available. Although previous studies have revealed a remarkable diversity of enzymes in the GH70 family in Lactic Acid Bacteria with regard to catalytic activities, domain organization patterns and 3D structures [9], the within-species diversity of these enzymes have not been explored. In this study, we defined subtypes of GH70 and GH32 domains based on subclades in the phylogeny and searched for correlations between the different subtypes and the presence/absence of glucan-binding domains, sequence motifs, strain distribution patterns and/or gene position. Below, we discuss the interplay between the mechanisms and selective forces that generate sequence divergence of enzymes involved in exopolysaccharide synthesis among strains within a species.

Our strategy was to first predict the catalytic activities of the GH70 domains and compare the relative fraction of catalytic domains within and among species. For the study, we selected a set of 38 strains of *A. kunkeei* as representatives of the genetic diversity of strains sampled thus far. The conservation of residues in the seven typical sequence motifs within each domain type is indicative of adaptive changes followed by strong purifying selection (*d*_*N*_*/d*_*S*_ < 0.1). We predicted that 75% of the 64 full-length enzymes with glucan-binding repeats contained a single GS domain, 17% contained a single BrS domain and 8% contained 2 catalytic domains. For comparison, a broader study of Lactic Acid Bacteria showed that 86% of the 203 glucan-binding GH70 enzymes contained a single GS domain, 8% contained a single BrS domain and 6% contained both a GS and a BrS domain [9]. Thus, the intra-species diversity of catalytic activities in *A. kunkeei* resembled the inter-species diversity of glucan-binding GH70 enzymes in Lactic Acid Bacteria.

However, a notable difference between the results of the two studies was that we predicted that as many as 30% of the 91 GH70 full-length enzymes in total in *A. kunkeei* lacked auxiliary glucan-binding domains (the so-called NGBs), as compared to only 2.4% of the 208 enzymes in total in the study based on a broader set of species [9]. Most of the few enzymes without glucan-binding domains were identified in species of the genus *Apilactobacillus* [9], suggesting that the taxonomic distribution pattern of the NGBs is more restricted than that of the glucan-binding enzymes. The catalytic activities of the NGBs are unknown, but two of the enzymes were tentatively classified as BrSs in a previous study based on the observation that the amino acid at position 8 of motif III was a glycine, as in the BrS domains [9]. However, the NGB clade was only distantly related to the BrS clade in the phylogenetic analyses presented here and elsewhere [9], making this classification doubtful. Interestingly, GH32 enzymes in *A. kunkeei* showed a similar pattern, where subtypes differed with regard to the number of glucan-binding domains, and enzymes classified into the S3 subtype were missing such domains altogether. Determining the catalytic activities of the different subtypes of GH70 and GH32 enzymes is an interesting avenue for future research.

The enzymes in the GH70 family may not only differ in their catalytic activities, but also in their ability to bind to the bacterial cell wall. We have shown previously that the NGBs in *A. kunkeei* strains A0901 and A1401 as well as the GS in *A. kunkeei* strain A1401 were detected in both the cell free supernatants and the cell pellet after exponential growth in fructose-rich media [17]. Likewise, several studies have detected GSs from *Leuconostoc* species in both the supernatant and the cell pellet following centrifugation of bacterial cells after growth in sucrose-supplemented media [62]. It has been assumed that the GH70 enzymes were secreted into the solution and became pelleted only because of their binding to dextran. However, growth of *Leuconostoc mesenteroides* showed that alternansucrase was enriched in the cell pellet despite the absence of dextran [63]. Subsequently, experimental studies of the dextransucrase DsrP in *L. mesenteroides* showed that the C-terminal domain mediated attachment to the bacterial cell wall [64]. Studies of glycosyltransferases from *Streptococcus* have also found extracellular and cell-associated glucansucrases and, in the latter, cell attachment was shown to be mediated by the C-terminal end of the protein [65, 66]. The binding abilities of these proteins may therefore depend on the C-terminal sequences. The proteins with a single BrS domain contained a domain of unknown function (DUF5776) and both the GS2 and the twin enzymes in *A. kunkeei* contained serine-rich repeat sequences at their C-terminal ends. Future studies are needed to determine if the C-terminal auxiliary domains mediate binding to the cell envelope or to exopolysaccharides. Since the non-glucan binding GH70 and GH32 enzymes lack auxiliary C-terminal domains, they are predicted to be secreted.

The GH70 enzymes with and without glucan-binding repeats are located at different positions in the genome, they are flanked by different genes and they have different origins and evolutionary histories. Since the NGBs were only identified in some *Apilactobacillus* species and their closest relatives, we infer that they have been vertically inherited in *A. kunkeei* from a recent common ancestor. In contrast, the GSs and BrSs in *A. kunkeei* clustered with otherwise distantly related bacteria, many of which are fructophilic Lactic Acid Bacteria, suggesting that they have been horizontally acquired. Furthermore, the presence of distinct subtypes within the GS and BrS clades, such as GS1, GS2 and GS3, suggest that they are paralogs and have expanded by a series of gene duplication events driven by selection for sub-functionalization. The gene for the GS1 enzyme represented the most frequently identified subtype, being present in as many as 90% of the strains. This gene was also positioned centrally in the gene cluster and was either flanked by genes for GS2 and BrS on the upstream side or by genes for glucan-binding GH32 enzymes on the downstream side. The co-location of genes for GH32 and GH70 enzymes could indicate an interaction between the enzymatic activities of proteins in these two families.

Interestingly, genes for the GS2 and the BrS domains mostly co-occurred in the same genome and were either encoded by two genes located next to each other or by a single gene encoding both domains. Twin GS2-BrS enzymes are rare and have so far only been found in *A. kunkeei* and in a few distantly related *Leuconostoc* species, but the twin enzymes in these species were not phylogenetically related, suggesting that the fusion events have occurred independently in the two lineages. We hypothesize that selection has favored the co-occurrence of the GS2 and BrS domains, perhaps because they act in concert during the synthesis of the exopolysaccharides. However, despite the co-occurrence of the two domains, they have not co-diverged. For example, the GS2 domains in the twin proteins of *A. kunkeei* clustered in the phylogeny, whereas the BrS domains of the same proteins segregated into two clades composed of two types of domain variants that catalyze distinct reactions, as inferred from previous experimental studies of one domain in each clade [21, 67]. Our hypothesis is that the sequences of the GS2 domains have been homogenized by repeated homologous recombination events, whereas the BrS domains of the same proteins have accumulated single nucleotide mutations or recombined in diverged fragments. The two subtypes may have been retained in the population due to selection for sub-functionalization and altered catalytic activities. An interesting focus for future research is to examine whether the repertoires of specific GH70/GH32 enzymes correlate with habitats and ecological niches.

The results presented here are consistent with previous findings which have shown that extracellular proteins change rapidly in gene sequence and display high rates of gene turnover [68, 69]. Since all *A. kunkeei* genomes are closed, the patchy distribution of genes for GH70 and GH32 proteins cannot be attributed to mis-assemblies, but reflect true gains and losses. The gene content dynamics may have been facilitated by the presence of IS3 transposons in the vicinity of genes for GH70 and GH32 proteins, along with transposon-containing plasmids and phagemids in the *A. kunkeei* population. Interestingly, glycosyl hydrolases have been shown to be the most common carbohydrate-active enzymes encoded by genes located on plasmids [70]. More specifically, a plasmid-borne gene for dextransucrase has been described in *Lactobacillus sakei* strain MN1 [71], although no genes for GH70 enzymes have as yet been detected on *A. kunkeei* plasmids or phagemids.

We wish to emphasize that the *d*_*N*_ and *d*_*S*_ values are used in this study as a proxy for sequence divergence and may not correspond to the true frequencies of nucleotide substitutions at nonsynonymous and synonymous sites, respectively. On the one hand, multiple gains, duplications and losses indicate that the genes being compared are paralogs, which may yield inflated *d*_*N*_ and *d*_*S*_ values. This might explain why most of the synonymous sites in the GH70 enzymes appear saturated for substitutions. On the other hand, high frequencies of homologous recombination events will bias the *d*_*N*_ and *d*_*S*_ estimates towards lower values than the true frequencies of nucleotide substitutions. These effects might explain why the sequences for the GH70 domains have exceptionally high *d*_*S*_ values, whereas the majority of the core genes have exceptionally low *d*_*S*_ values.

In addition, genes for the GH70 enzymes may have escaped from the homogenizing effects of homologous recombination due to the strain-specific gene order structures for the GH70 and GH32 enzymes, in line with the hypothesis that rampant gene gain and loss generate barriers to recombination and allow sequence divergence levels to increase [72–74]. Beyond a certain threshold level, homogenization by homologous recombination may no longer be possible, thereby further contributing to divergent evolutionary trajectories. Intra-genic recombination events across short stretches of nearly identical sequences may still occur, and very long recombination tracts that span across the entire genomic region would not be affected. Our study showed that both of these types of events have occurred in the genomic region for GH70 and GH32 enzymes.

The *A. kunkeei* isolates have been sorted into six phylogroups, named A-F, as inferred from a maximum likelihood tree based on concatenated core protein alignments [18, 19]. Phylogroups A-C showed average nucleotide sequence identity values > 95% [18, 19], confirming that they belong to the same species according to the criteria normally used for species designations. The relative effects of recombination versus single nucleotide substitutions (*r/m*) to the divergence of the core genome in the *A. kunkeei* phylogroup A and phylogroup B-C strains were estimated to 1.6 and 5.2, respectively, which is similar to previous estimates of *r/m* ratios of about 2–3 [75, 76]. The high influence of homologous recombination to the homogenization of the core genes provides an explanation for the clustering of *A. kunkeei* strains into distinct phylogroups [18, 19]. The neutral model for bacterial diversification suggests that homologous recombination can maintain cohesive population structures even in the absence of selection for genomes with ANI values > 95% and *r/m* values > 0.33 [77]. Thus, the clustering of the phylogroup A-C strains may be explained by neutral processes alone, although this does not exclude that ecological cohesiveness [78] may also have played a role.

Strains assigned to phylogroups E and F were excluded from the recombination analyses because they showed ANI values < 93% compared to the phylogroup A-C strains [18]. This suggests that they have diverged beyond the level needed to maintain a cohesive structure and should be classified as distinct species. Indeed, it has been suggested that a continuum exists from very closely related strains where recombination continuously homogenizes most of the genes to more distantly related strains in which frequent gene gain and loss reduces the incidence of recombination events, which in the long-term could lead to isolation and speciation [79]. Strain A1404 from phylogroup E contained no genes for GH70 enzymes and strain A1001 contained a single gene for a GH70 enzyme that was tentatively classified as GS1, but is rather distantly related to the other GS1 enzymes. Further sampling and studies of early diverging *A. kunkeei* strains may provide more information about barriers to recombination that enable strains to segregate into distinct species even within the same growth niche. At the same time, such studies may help identify GH70 enzymes with novel catalytic activities.

Taken together, our study suggests that each gene in the *A. kunkeei* genome has its own unique history due to the interplay between gene content divergence and gene sequence homogenization by recombination events. For this reason, future attempts to define the taxonomy of *A. kunkeei* strains and related species should rely on multi-gene trees that integrate the different evolutionary histories of each gene. For a mechanistic understanding of the diversification patterns, phylogenetic inferences should be complemented with recombination detection studies.

## Supplementary Information

Additional file 1: Table S1. List of the NCBI accession numbers and names of the 106 A. kunkeei genomes, with the download date and time from NCBI. Table S2. List of the 38 groups of A. kunkeei genomes and the representative strains compared to the representative strains from Dyrhage et al. [18]. Table S3. List of GH70 genes in the 106 A. kunkeei genomes (strain, gene type, ID, position and length) and the domains that are contained in these genes. Table S4. List of GH32 genes in the 106 A. kunkeei genomes (strain, gene type, ID, position and length) and the domains that are contained in these genes. Table S5. PCR primer sequence information, including target strain and gene, locus tags, locus ID and primer sequence, length and Tm. Table S6. Sequence motifs in each GH70 domain. A) Gene ID and type and motif number, sequence and position. B) Summary of sequence motifs in each gene type. Table S7. Sequence motifs in each GH32 domain. A) Gene ID and type and motif number, sequence and position. B) Summary of sequence motifs in each gene type. Table S8. List of GH70 genes in bacterial species other than A. kunkeei (gene ID, gene type and motif number, position and length). Table S9. Synonymous (dS) and nonsynonymous (dN) substitution frequencies of gene sequences for GH70 domains compared to the mean dN and dS and median dN/dS for core genes in pairwise comparisons of A. kunkeei strains. Table S10. List of recombination events predicted by RDP5. Table S11. ClonalFrameML parameter estimates for phylogroup A and phylogroup B-C.

Additional file 2: Figure S1. Sanger sequencing chromatograms of the PCR products of primer pairs targeting GH genes A) A1401_12760-70 (GS2 and BrS segments), B) H3B111M_12570-80 (BrS), C) FHON2_13560-70 (GS2), D) H3B209X_13360-70 (GS2), E) G0102_12710-20 (GS1). Putative stop codons are highlighted in grey, and differences with the original sequence are highlighted in red. R = ruler, - = negative control. Figure S2. Phylogenetic relationships of GH70 and GH32 domains. Phylogenetic trees were inferred from the amino acid alignments of the A) 907 core proteins, B) 97 GH70 domains plus four outgroups and C) 25 GH32 domains in 38 representative strains of A. kunkeei. Strain names and locus tags are colored by phylogroup designations according to the core protein phylogeny by Dyrhage et al. [18]. The catalytic activities of protein domains with experimentally confirmed functions are indicated in the yellow boxes. Proteins annotated as GTFB in Limosilactobacillus reuteri and Limosilactobacillus fermentum were used as outgroups in the GH70 domain phylogeny. The trees were inferred with maximum likelihood methods. Numbers on nodes show bootstrap support values ≥50. Figure S3. Possible domain organizations of the GH70 and GH32 family proteins. Organization of domains and sequence repeats in a subset of A) GH70 and B) GH32 enzymes with and without glucan-binding repeats in the 38 reference A. kunkeei strains. All domains and repeats are based on InterProScan predictions, except for signal peptides, which are based on SignalP6 (slow mode) predictions; glucan-binding domains, which are based on AlphaFold predictions, and serine-rich repeats, which are based on protein sequence. GS = glucansucrases, BrS = branching sucrases, NGB = non-glucan binding GH70, PTL = proteins with the N-terminal segment of a GH70 domain, S1-3 = GH32 subtypes, of which S3 lacks glucan-binding domains. Figure S4. Pairwise BLASTN comparisons of a 40 kb segment of the A. kunkeei genome with the genes coding for GH70 and GH32 enzymes. The order of strains is based on the core protein phylogeny shown in Figure S2A. Strain names are colored by phylogroup designations. Forward BLASTN matches are colored in grey and reverse matches, in red. GH70 and GH32 are colored depending on the subtype, and transposons are colored in black. Figure S5. Pairwise BLASTN comparisons of a 40 kb segment of the A. kunkeei genome with the genes coding for the NGB enzymes. The order of strains is based on the core protein phylogeny shown in Figure S2A. Strain names are colored by phylogroup designations. Forward BLASTN matches are colored in grey and reverse matches, in red. NGB is colored in dark blue-green, phage genes in purple, ABC transporters in green, CRISPR genes in brown and transposons in black. Figure S6. Phylogenetic relationships of GH70 domains in bacteria. Phylogenetic trees were inferred with the maximum likelihood method from the amino acid alignments of GH70 domains in members of the Lactobacillaceae, Streptococcaceae and Enterococcaceae. The clades containing GSs, BrSs and NGBs in the reference A. kunkeei strains and A. apinorum are schematically indicated by circles colored in orange. Species names and gene IDs are colored by family designations. The catalytic activities of protein domains with experimentally confirmed functions are indicated by AS (alternansucrase), DS (dextransucrase), MS (mutansucrase), RS (reuteransucrase) or BrS labels, all marked in yellow. Proteins annotated as GTFB in Limosilactobacillus reuteri and Limosilactobacillus fermentum were used as outgroups. The subtypes within which the domains cluster (GS, BrS, NGB or GTFB) are indicated by dotted rectangles. Numbers on nodes show bootstrap support values ≥50. Figure S7. Comparison of GH70 domain tree topologies. Comparisons of A, B) tree topologies and C, D) quartet-based internode certainty scores for GH70 and core proteins. Phylogenetic trees and were inferred based on the amino acid alignments of the A) GS1 and B) NGB domains in the two subsets of A. kunkeei strains that contain genes for these proteins. The tree topologies were compared to the topologies obtained from phylogenetic inferences based on 907 core proteins in the two subsets of A. kunkeei strains, respectively. The trees were inferred with the maximum likelihood method. Bootstrap support values of 100% and ≥ 95% are shown as black and grey dots, respectively. Quartet-based internode certainty score comparisons of the core protein phylogenies and the C) GS1 and D) NGB domain phylogenies. The branch values display the lowest quartet internode certainty (LQ-IC), the quadripartition internode certainty (QP-IC) and the extended quadripartition internode certainty (EQP-IC) scores, where values close to 1 indicate support of the internal branch, values close to 0 indicate high levels of incongruence and values close to -1 contest it. Branches with LQ-IC scores close to 1 are highlighted. The GH70 and core protein input phylogenies were unrooted in the QuartetScore analysis, and the resulting trees were midpoint rooted for visualization purposes. Strain names and locus tags are colored by phylogroup designations. Figure S8. Recombination breakpoint plot, showing the genes in the genomic region for GH70 and GH32 family proteins. The lines at the top of the plot represent predicted breakpoints. The dashed red line represents the join point between the two alignments. Gaps are shown as grey rectangles. The continuous black line represents the number of predicted breakpoints per 200 nt windows along the alignment. The two shaded areas behind the black line represent the confidence intervals (CIs), where the strongly-shaded area represents the 95% CI and the faintly-shaded area represents the 99% CI. Green triangles represent parts of the alignment where the number of predicted breakpoints is above the 95% CI, if less saturated, or the 99% CI, if more saturated. The coding sequences along the alignment are shown at the bottom. Figure S9. ClonalFrameML plot showing recombination along the A. kunkeei chromosome for A) phylogroup A and B) phylogroups B-C. Dark blue = predicted recombination. Light blue = no change. White, yellow and orange represent the degree of homoplasy, with white being the lowest and orange, the highest.

## Data Availability

The datasets generated and analysed during the current study are available in the SciLifelab Data Repository, 10.17044/scilifelab.c.7884695. The custom scripts used for this project are available at 10.5281/zenodo.17985948.

## Abbreviations

GH70: Family 70 glycosyl hydrolase
GH32: Family 32 glycosyl hydrolase
HoPS: Homopolymers
HePS: Heteropolymers
α-1,X: Type of α-glycosydic linkage between glucose subunits, from carbon 1 of the glucose molecule to carbon X of the next
GS: Glucansucrase
BrS: Branching sucrase
GBD: Glucan-binding domain
NGB: Non-glucan-binding
PTL: Partial
S1-S3: Subtypes 1–3
CD1-2: Catalytic domain 1–2
*ν*: Sequence divergence
*δ*: Size of the recombination tracts (bp)
*R/**θ*: Frequency of recombination relative to the rate of mutation
*r/m*: Ratio of the relative effect of recombination and mutation to the sequence divergences
*d*_*N*_*/d*_*S*_: Ratio of nonsynonymous to synonymous substitutions
ANI: Average nucleotide identity
PAE: Predicted aligned error
PCR: Polymerase chain reaction
SNP: Single nucleotide polymorphism
CDS: Coding sequence
ORI: Origin of replication
