# Supplementary material for: Diversity of enzymes for exopolysaccharide synthesis in the fructophilic honeybee symbiont Apilactobacillus kunkeei
Source: BMC Microbiol. 2026 Jan 12;26:62. doi: 10.1186/s12866-025-04680-3 (PMC12849098; doi:10.1186/s12866-025-04680-3)
Supplement: Supplementary file 2 — Additional file 2: Figure S1. Sanger sequencing chromatograms of the PCR products of primer pairs targeting GH genes A) A1401_12760-70 (GS2 and BrS segments), B) H3B111M_12570-80 (BrS), C) FHON2_13560-70 (GS2), D) H3B209X_13360-70 (GS2), E) G0102_12710-20 (GS1). Putative stop codons are highlighted in grey, and differences with the original sequence are highlighted in red. R = ruler, - = negative control. Figure S2. Phylogenetic relationships of GH70 and GH32 domains. Phylogenetic trees were inferred from the amino acid alignments of the A) 907 core proteins, B) 97 GH70 domains plus four outgroups and C) 25 GH32 domains in 38 representative strains of A. kunkeei. Strain names and locus tags are colored by phylogroup designations according to the core protein phylogeny by Dyrhage et al. [18]. The catalytic activities of protein domains with experimentally confirmed functions are indicated in the yellow boxes. Proteins annotated as GTFB in Limosilactobacillus reuteri and Limosilactobacillus fermentum were used as outgroups in the GH70 domain phylogeny. The trees were inferred with maximum likelihood methods. Numbers on nodes show bootstrap support values ≥50. Figure S3. Possible domain organizations of the GH70 and GH32 family proteins. Organization of domains and sequence repeats in a subset of A) GH70 and B) GH32 enzymes with and without glucan-binding repeats in the 38 reference A. kunkeei strains. All domains and repeats are based on InterProScan predictions, except for signal peptides, which are based on SignalP6 (slow mode) predictions; glucan-binding domains, which are based on AlphaFold predictions, and serine-rich repeats, which are based on protein sequence. GS = glucansucrases, BrS = branching sucrases, NGB = non-glucan binding GH70, PTL = proteins with the N-terminal segment of a GH70 domain, S1-3 = GH32 subtypes, of which S3 lacks glucan-binding domains. Figure S4. Pairwise BLASTN comparisons of a 40 kb segment of the A. kunkeei genome with the genes codi [file 12866_2025_4680_MOESM2_ESM.pdf]

A

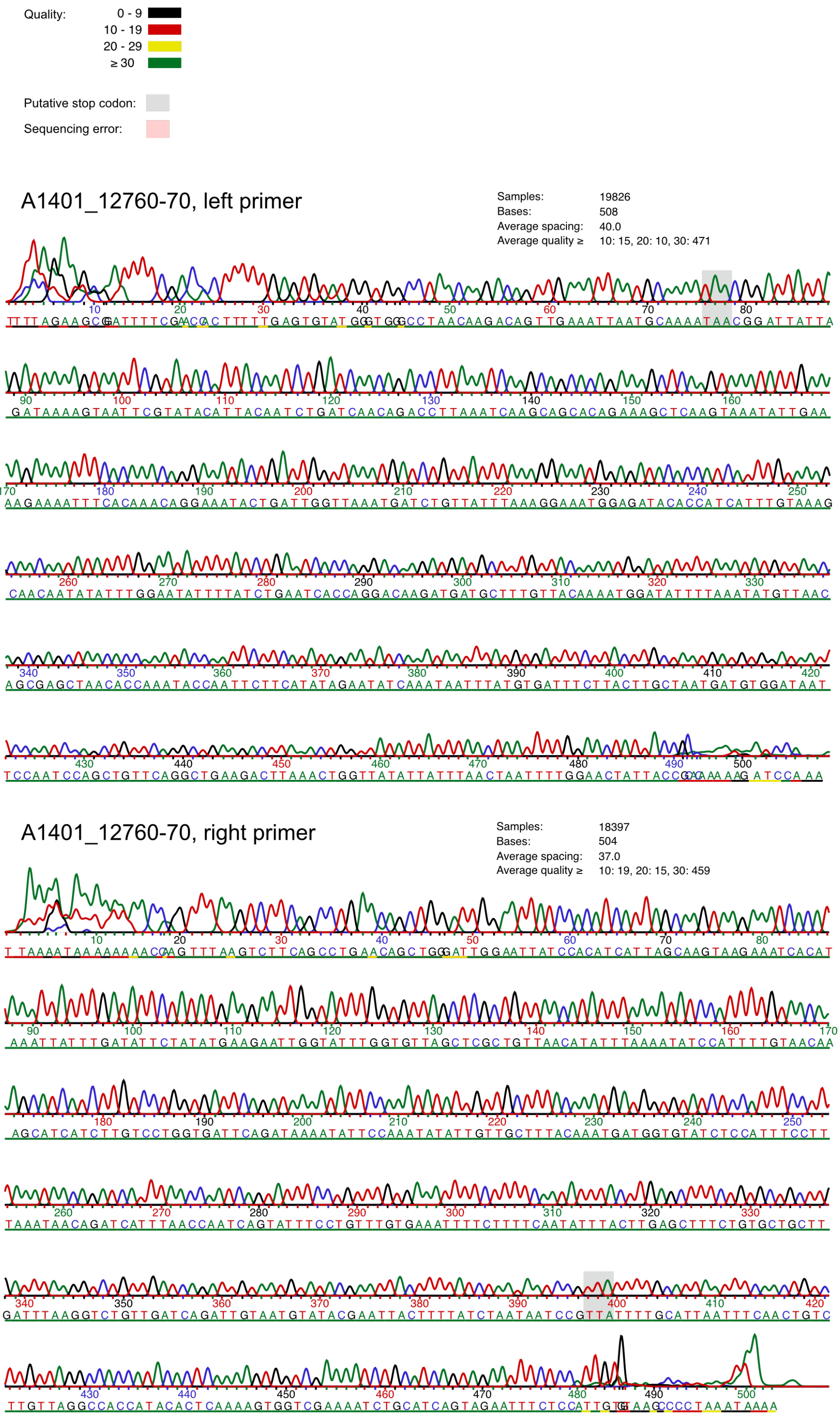

Quality:
0 - 9
10 - 19
20 - 29
≥ 30
Putative stop codon:
Sequencing error:
A1401_12760-70, left primer
Samples: 19826
Bases: 508
Average spacing: 40.0
Average quality ≥ 10: 15, 20: 10, 30: 471
A1401_12760-70, right primer
Samples: 18397
Bases: 504
Average spacing: 37.0
Average quality ≥ 10: 19, 20: 15, 30: 459


Figure S1A

B

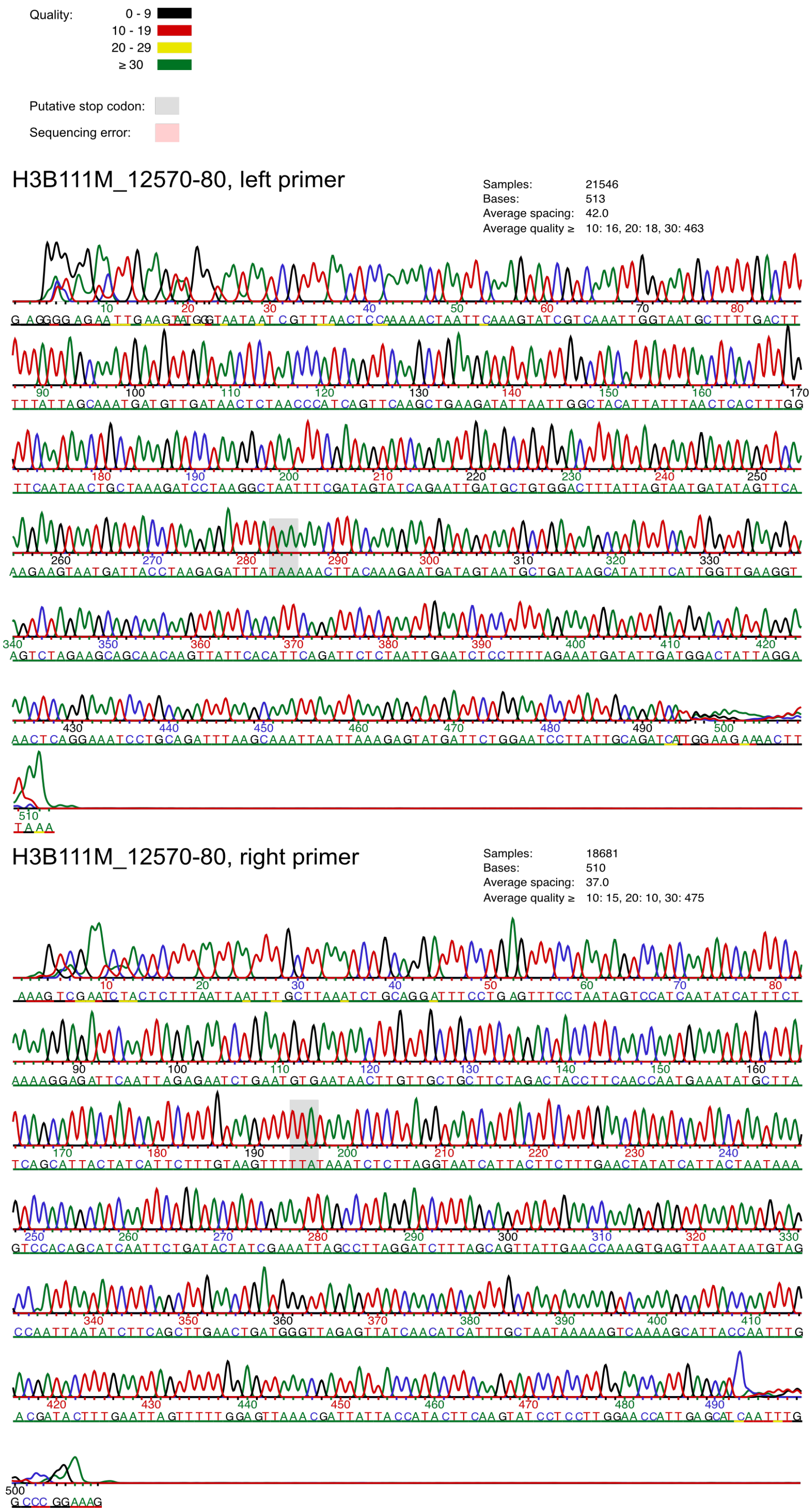
Quality:
0 - 9
10 - 19
20 - 29
≥ 30
Putative stop codon:
Sequencing error:
H3B111M_12570-80, left primer
Samples: 21546
Bases: 513
Average spacing: 42.0
Average quality ≥ 10: 16, 20: 18, 30: 463
H3B111M_12570-80, right primer
Samples: 18681
Bases: 510
Average spacing: 37.0
Average quality ≥ 10: 15, 20: 10, 30: 475

Figure S1B

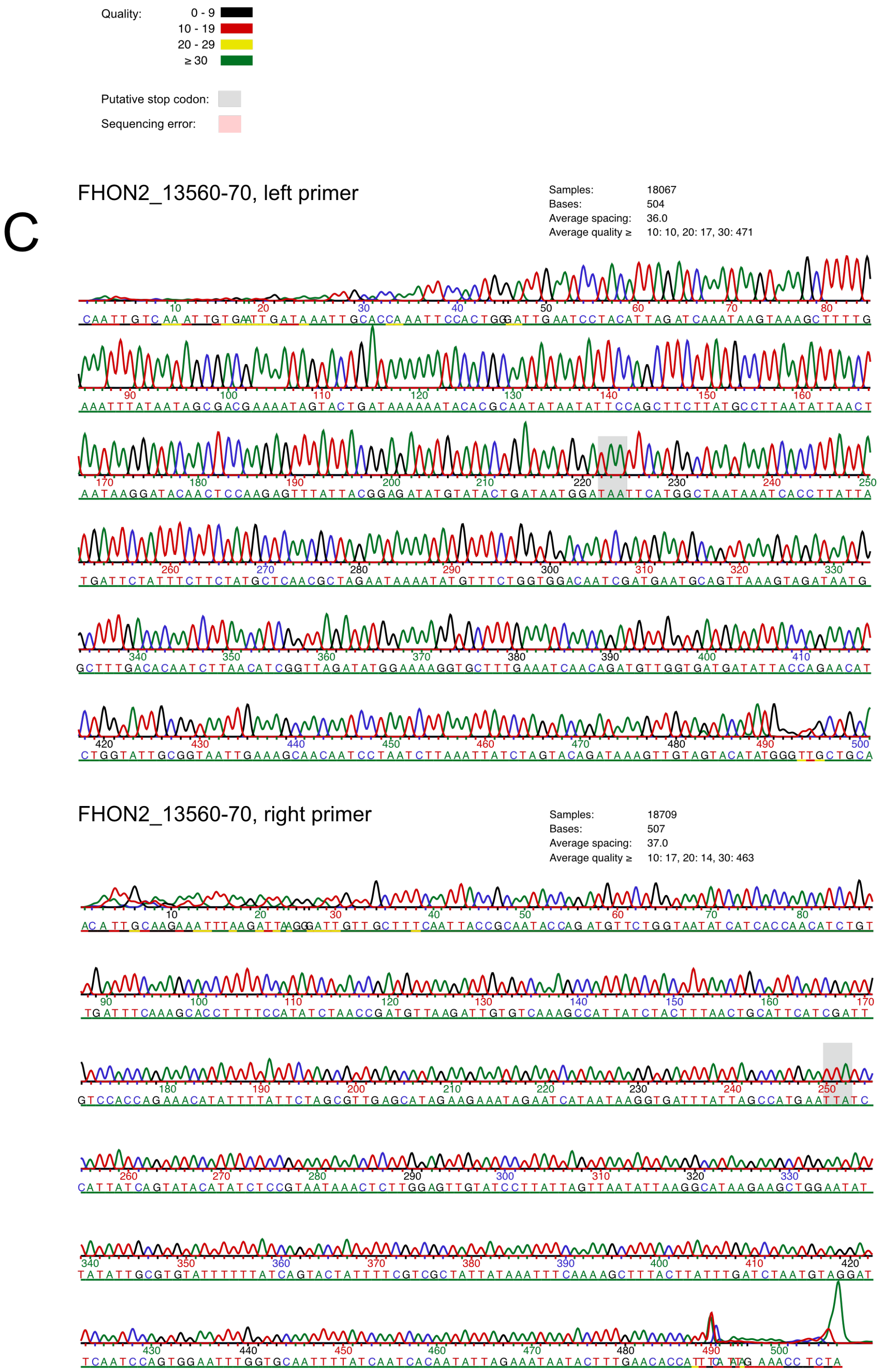

Quality:
0 - 9
10 - 19
20 - 29
≥ 30
Putative stop codon:
Sequencing error:
C
FHON2_13560-70, left primer
Samples: 18067
Bases: 504
Average spacing: 36.0
Average quality ≥ 10: 10, 20: 17, 30: 471
FHON2_13560-70, right primer
Samples: 18709
Bases: 507
Average spacing: 37.0
Average quality ≥ 10: 17, 20: 14, 30: 463


Figure S1C

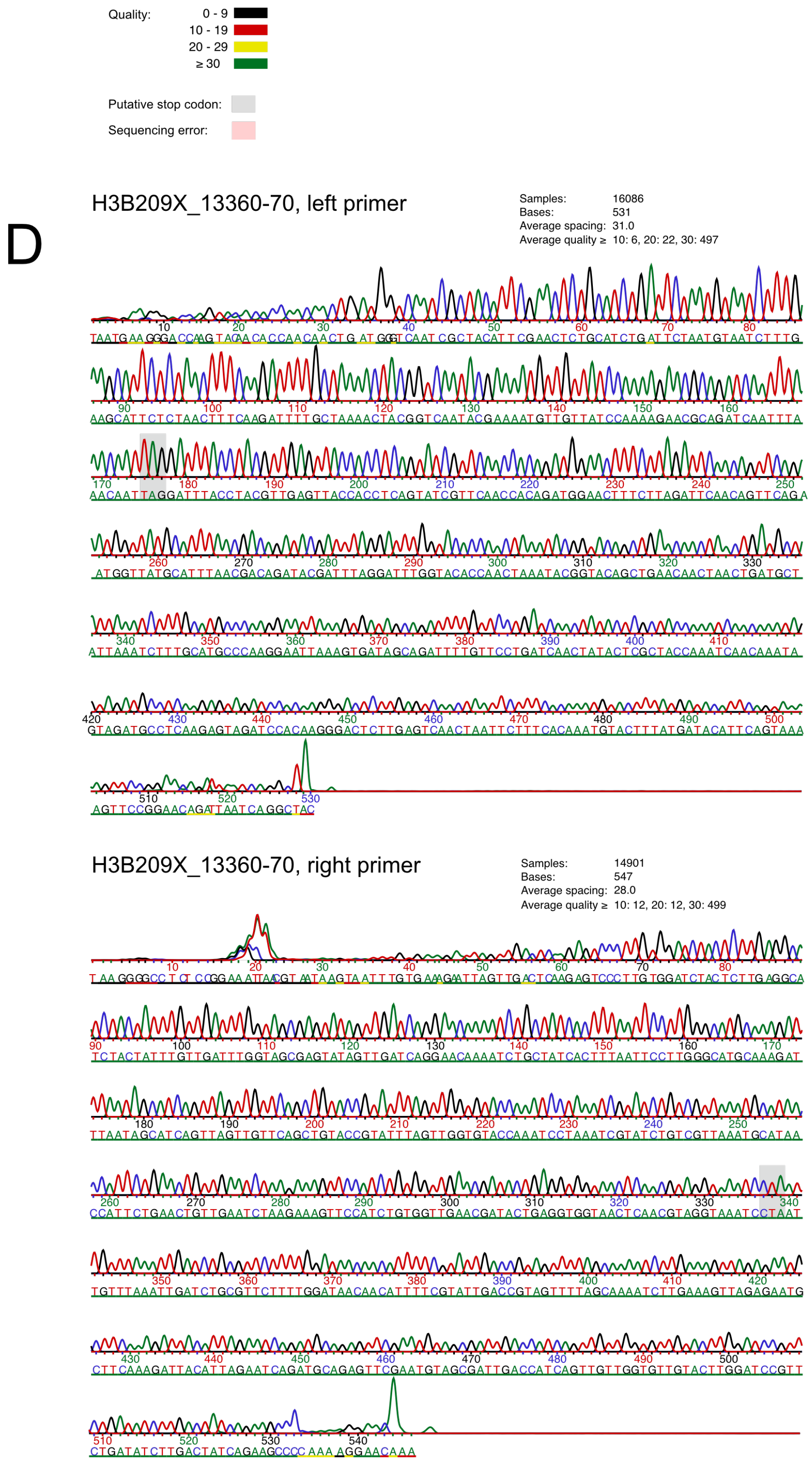
Quality:
0 - 9
10 - 19
20 - 29
≥ 30
Putative stop codon:
Sequencing error:
D
H3B209X_13360-70, left primer
Samples: 16086
Bases: 531
Average spacing: 31.0
Average quality ≥ 10: 6, 20: 22, 30: 497
H3B209X_13360-70, right primer
Samples: 14901
Bases: 547
Average spacing: 28.0
Average quality ≥ 10: 12, 20: 12, 30: 499

Figure S1D

E

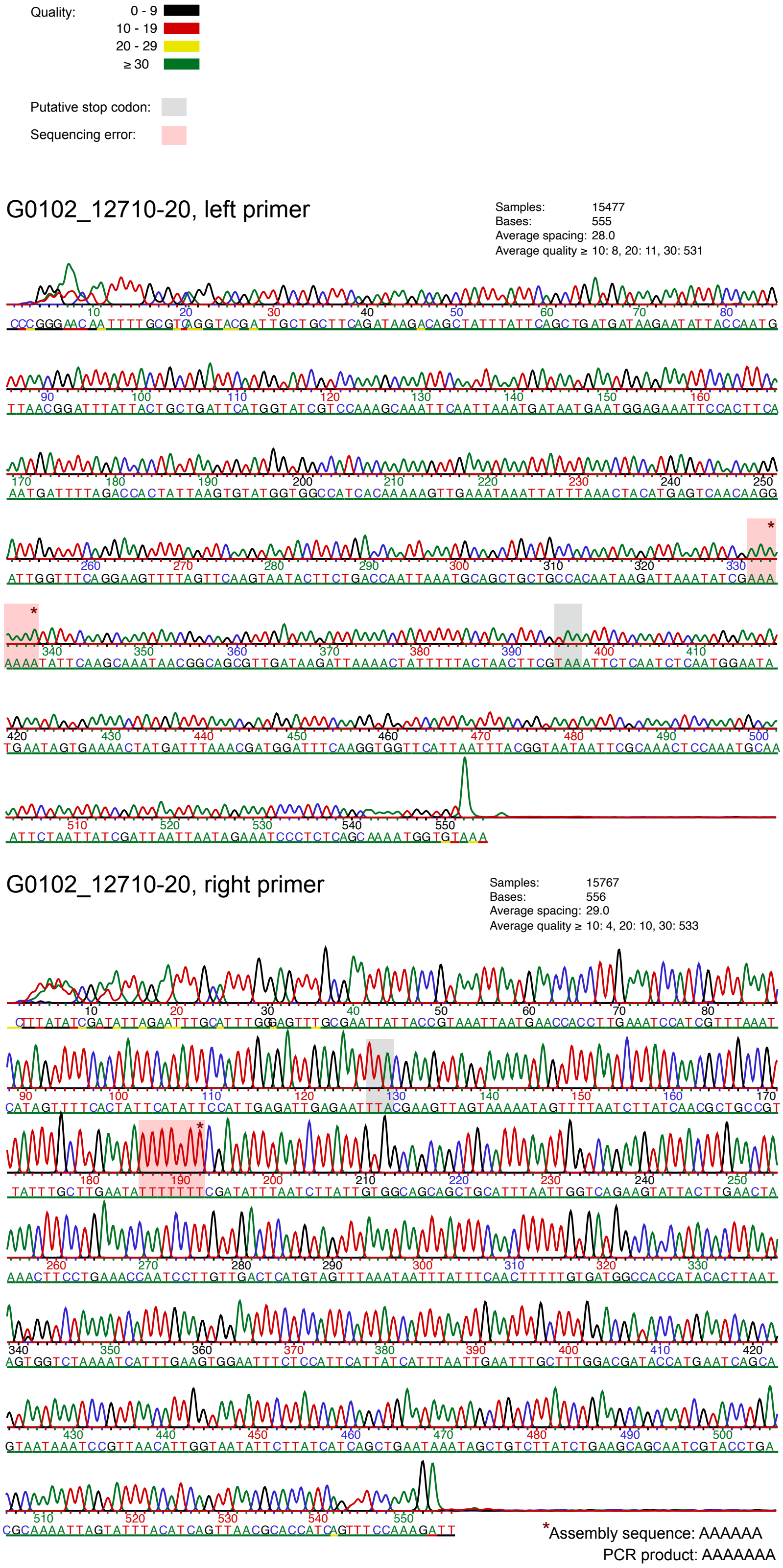
Quality:
0 - 9
10 - 19
20 - 29
≥ 30
Putative stop codon:
Sequencing error:
G0102_12710-20, left primer
Samples: 15477
Bases: 555
Average spacing: 28.0
Average quality ≥ 10: 8, 20: 11, 30: 531
G0102_12710-20, right primer
Samples: 15767
Bases: 556
Average spacing: 29.0
Average quality ≥ 10: 4, 20: 10, 30: 533
*Assembly sequence: AAAAAA
PCR product: AAAAAAA

Figure S1E

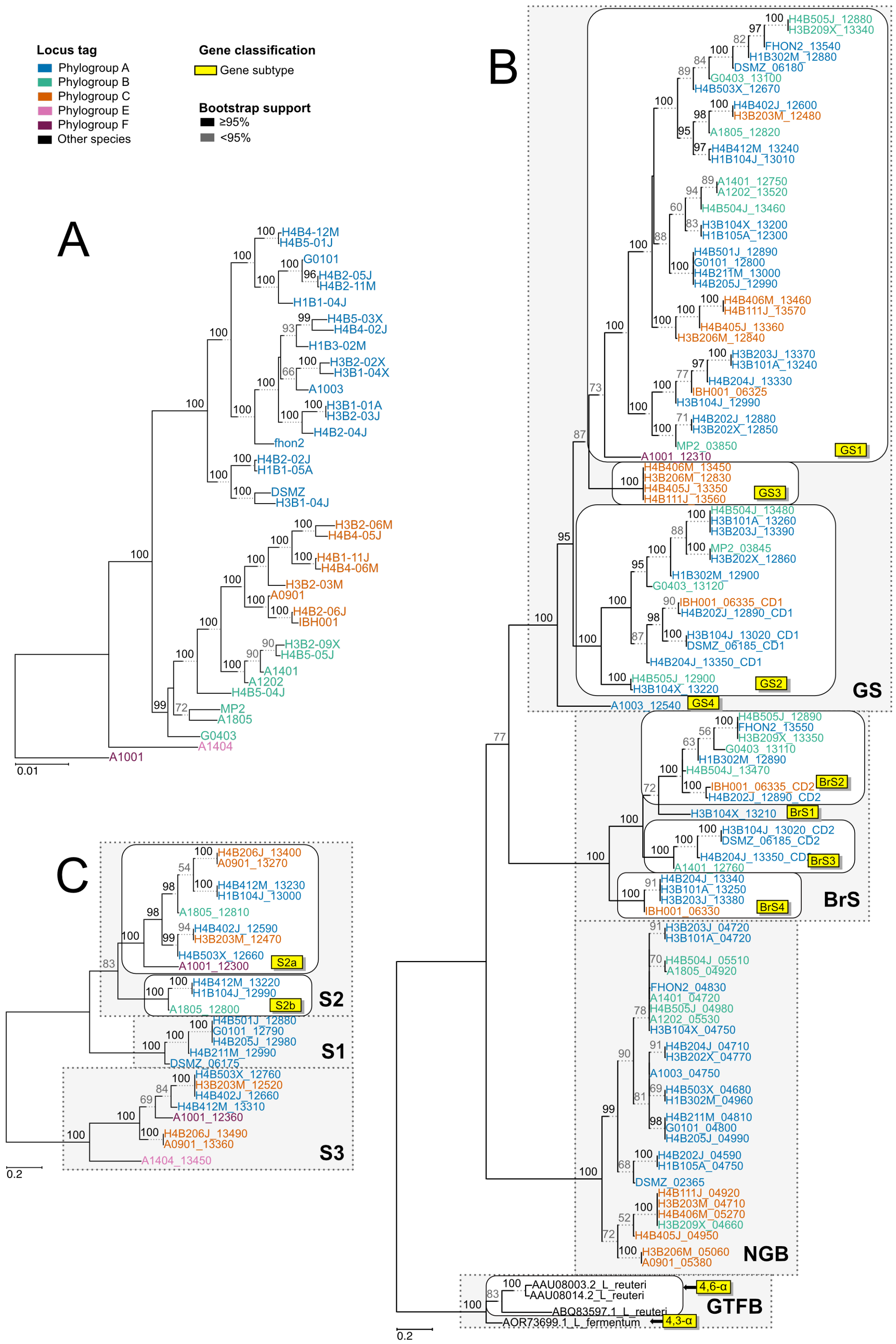

Locus tag
Phylogroup A
Phylogroup B
Phylogroup C
Phylogroup E
Phylogroup F
Other species
Gene classification
Gene subtype
Bootstrap support
≥95%
<95%
A
H4B4-12M
H4B5-01J
G0101
H4B2-05J
H4B2-11M
H1B1-04J
H4B5-03X
H4B4-02J
H1B3-02M
H3B2-02X
H3B1-04X
A1003
H3B1-01A
H3B2-03J
H4B2-04J
fhon2
H4B2-02J
H1B1-05A
DSMZ
H3B1-04J
H3B2-06M
H4B4-05J
H4B1-11J
H4B4-06M
H3B2-03M
A0901
H4B2-06J
IBH001
H3B2-09X
H4B5-05J
A1401
A1202
H4B5-04J
MP2
A1805
G0403
A1404
A1001
0.01
B
H4B505J_12880
H3B209X_13340
FHON2_13540
H1B302M_12880
DSMZ_06180
G0403_13100
H4B503X_12670
H4B402J_12600
H3B203M_12480
A1805_12820
H4B412M_13240
H1B104J_13010
A1401_12750
A1202_13520
H4B504J_13460
H3B104X_13200
H1B105A_12300
H4B501J_12890
G0101_12800
H4B211M_13000
H4B205J_12990
H4B406M_13460
H4B111J_13570
H4B405J_13360
H3B206M_12840
H3B203J_13370
H3B101A_13240
H4B204J_13330
IBH001_06325
H3B104J_12990
H4B202J_12880
H3B202X_12850
MP2_03850
A1001_12310
GS1
H4B406M_13450
H3B206M_12830
H4B405J_13350
H4B111J_13560
GS3
H4B504J_13480
H3B101A_13260
H3B203J_13390
MP2_03845
H3B202X_12860
H1B302M_12900
G0403_13120
IBH001_06335_CD1
H4B202J_12890_CD1
H3B104J_13020_CD1
DSMZ_06185_CD1
H4B204J_13350_CD1
H4B505J_12900
H3B104X_13220
GS2
A1003_12540
GS4
GS
H4B505J_12890
FHON2_13550
H3B209X_13350
G0403_13110
H1B302M_12890
H4B504J_13470
BrS2
IBH001_06335_CD2
H4B202J_12890_CD2
H3B104X_13210
BrS1
H3B104J_13020_CD2
DSMZ_06185_CD2
H4B204J_13350_CD
BrS3
A1401_12760
H4B204J_13340
H3B101A_13250
H3B203J_13380
IBH001_06330
BrS4
BrS
H3B203J_04720
H3B101A_04720
H4B504J_05510
A1805_04920
FHON2_04830
A1401_04720
H4B505J_04980
A1202_05530
H3B104X_04750
H4B204J_04710
H3B202X_04770
A1003_04750
H4B503X_04680
H1B302M_04960
H4B211M_04810
G0101_04800
H4B205J_04990
H4B202J_04590
H1B105A_04750
DSMZ_02365
H4B111J_04920
H3B203M_04710
H4B406M_05270
H3B209X_04660
H4B405J_04950
H3B206M_05060
A0901_05380
NGB
AAU08003.2_L_reuteri
AAU08014.2_L_reuteri
ABQ83597.1_L_reuteri
AOR73699.1_L_fermentum
4,6-α
4,3-α
GTFB
0.2
C
H4B206J_13400
A0901_13270
H4B412M_13230
H1B104J_13000
A1805_12810
H4B402J_12590
H3B203M_12470
H4B503X_12660
A1001_12300
S2a
H4B412M_13220
H1B104J_12990
A1805_12800
S2b
S2
H4B501J_12880
G0101_12790
H4B205J_12980
H4B211M_12990
DSMZ_06175
S1
H4B503X_12760
H3B203M_12520
H4B402J_12660
H4B412M_13310
A1001_12360
H4B206J_13490
A0901_13360
A1404_13450
S3
0.2


Figure S2

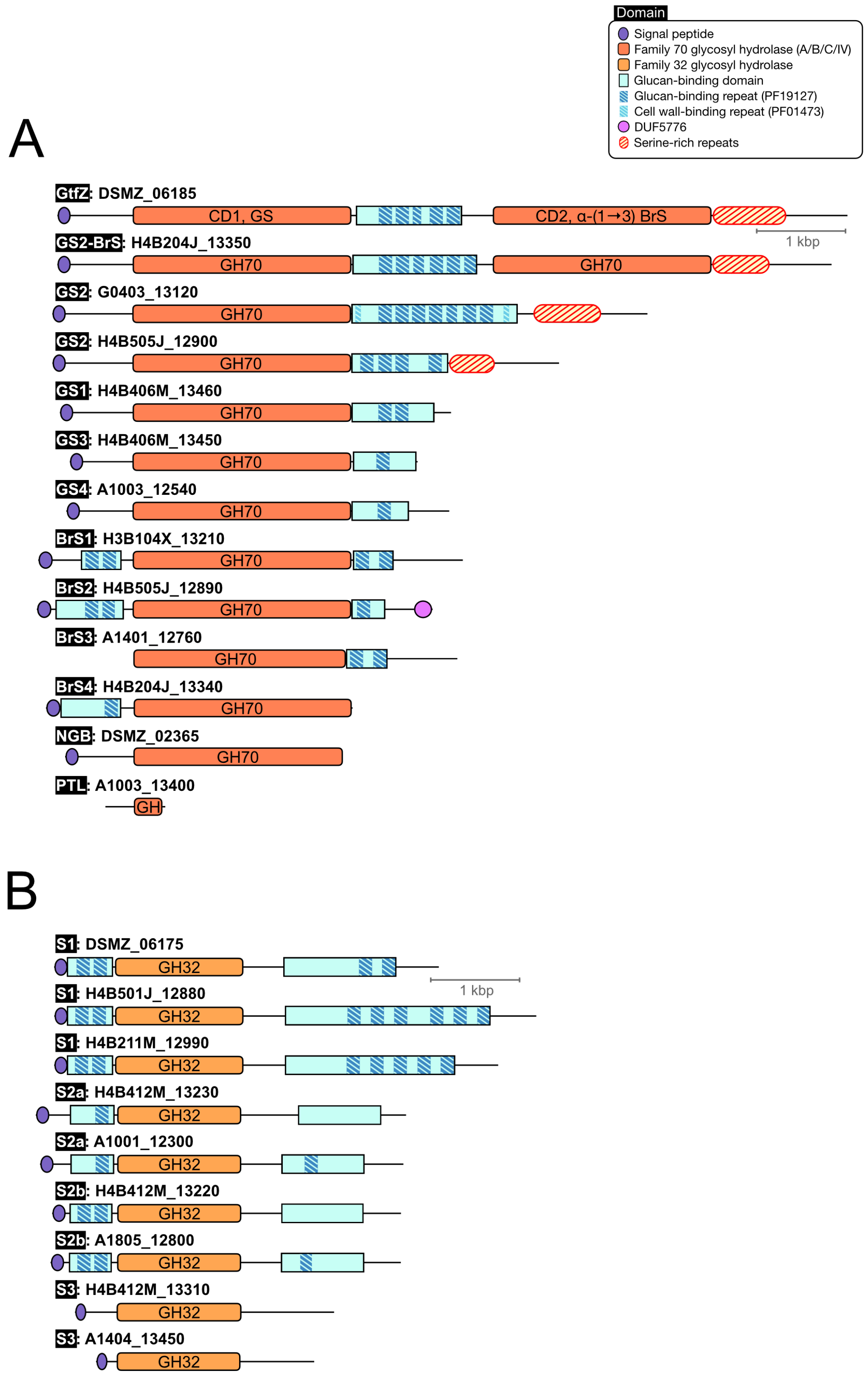
Domain
Signal peptide
Family 70 glycosyl hydrolase (A/B/C/IV)
Family 32 glycosyl hydrolase
Glucan-binding domain
Glucan-binding repeat (PF19127)
Cell wall-binding repeat (PF01473)
DUF5776
Serine-rich repeats
A
GtfZ: DSMZ_06185
CD1, GS
CD2, α-(1→3) BrS
1 kbp
GS2-BrS: H4B204J_13350
GH70
GH70
GS2: G0403_13120
GH70
GS2: H4B505J_12900
GH70
GS1: H4B406M_13460
GH70
GS3: H4B406M_13450
GH70
GS4: A1003_12540
GH70
BrS1: H3B104X_13210
GH70
BrS2: H4B505J_12890
GH70
BrS3: A1401_12760
GH70
BrS4: H4B204J_13340
GH70
NGB: DSMZ_02365
GH70
PTL: A1003_13400
GH
B
S1: DSMZ_06175
GH32
1 kbp
S1: H4B501J_12880
GH32
S1: H4B211M_12990
GH32
S2a: H4B412M_13230
GH32
S2a: A1001_12300
GH32
S2b: H4B412M_13220
GH32
S2b: A1805_12800
GH32
S3: H4B412M_13310
GH32
S3: A1404_13450
GH32

Figure S3

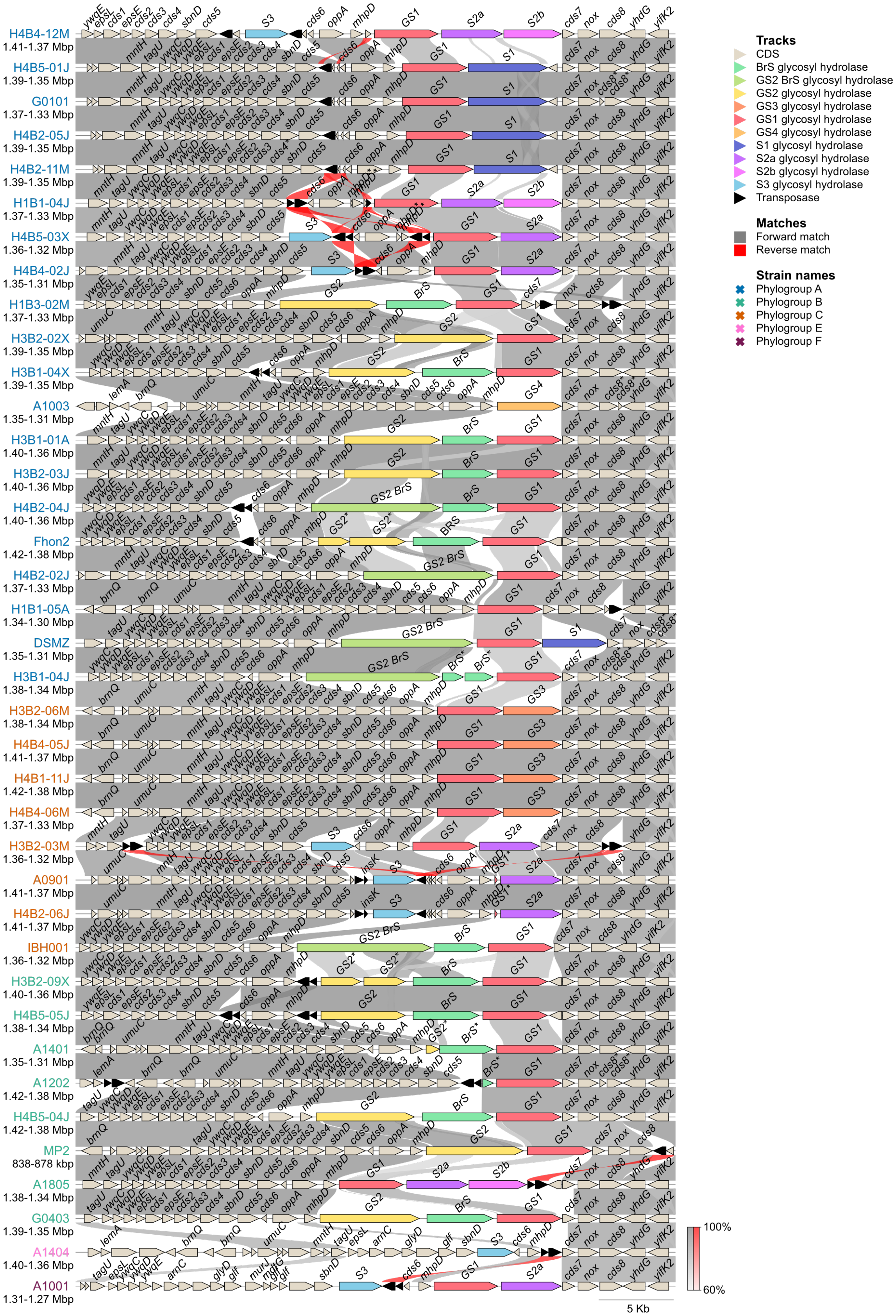
Tracks
CDS
BrS glycosyl hydrolase
GS2 BrS glycosyl hydrolase
GS2 glycosyl hydrolase
GS3 glycosyl hydrolase
GS1 glycosyl hydrolase
GS4 glycosyl hydrolase
S1 glycosyl hydrolase
S2a glycosyl hydrolase
S2b glycosyl hydrolase
S3 glycosyl hydrolase
Transposase
Matches
Forward match
Reverse match
Strain names
Phylogroup A
Phylogroup B
Phylogroup C
Phylogroup E
Phylogroup F
H4B4-12M 1.41-1.37 Mbp
H4B5-01J 1.39-1.35 Mbp
G0101 1.37-1.33 Mbp
H4B2-05J 1.39-1.35 Mbp
H4B2-11M 1.39-1.35 Mbp
H1B1-04J 1.37-1.33 Mbp
H4B5-03X 1.36-1.32 Mbp
H4B4-02J 1.35-1.31 Mbp
H1B3-02M 1.37-1.33 Mbp
H3B2-02X 1.39-1.35 Mbp
H3B1-04X 1.39-1.35 Mbp
A1003 1.35-1.31 Mbp
H3B1-01A 1.40-1.36 Mbp
H3B2-03J 1.40-1.36 Mbp
H4B2-04J 1.40-1.36 Mbp
Fhon2 1.42-1.38 Mbp
H4B2-02J 1.37-1.33 Mbp
H1B1-05A 1.34-1.30 Mbp
DSMZ 1.35-1.31 Mbp
H3B1-04J 1.38-1.34 Mbp
H3B2-06M 1.38-1.34 Mbp
H4B4-05J 1.41-1.37 Mbp
H4B1-11J 1.42-1.38 Mbp
H4B4-06M 1.37-1.33 Mbp
H3B2-03M 1.36-1.32 Mbp
A0901 1.41-1.37 Mbp
H4B2-06J 1.41-1.37 Mbp
IBH001 1.36-1.32 Mbp
H3B2-09X 1.40-1.36 Mbp
H4B5-05J 1.38-1.34 Mbp
A1401 1.35-1.31 Mbp
A1202 1.42-1.38 Mbp
H4B5-04J 1.42-1.38 Mbp
MP2 838-878 kbp
A1805 1.38-1.34 Mbp
G0403 1.39-1.35 Mbp
A1404 1.40-1.36 Mbp
A1001 1.31-1.27 Mbp
100%
60%
5 Kb

Figure S4

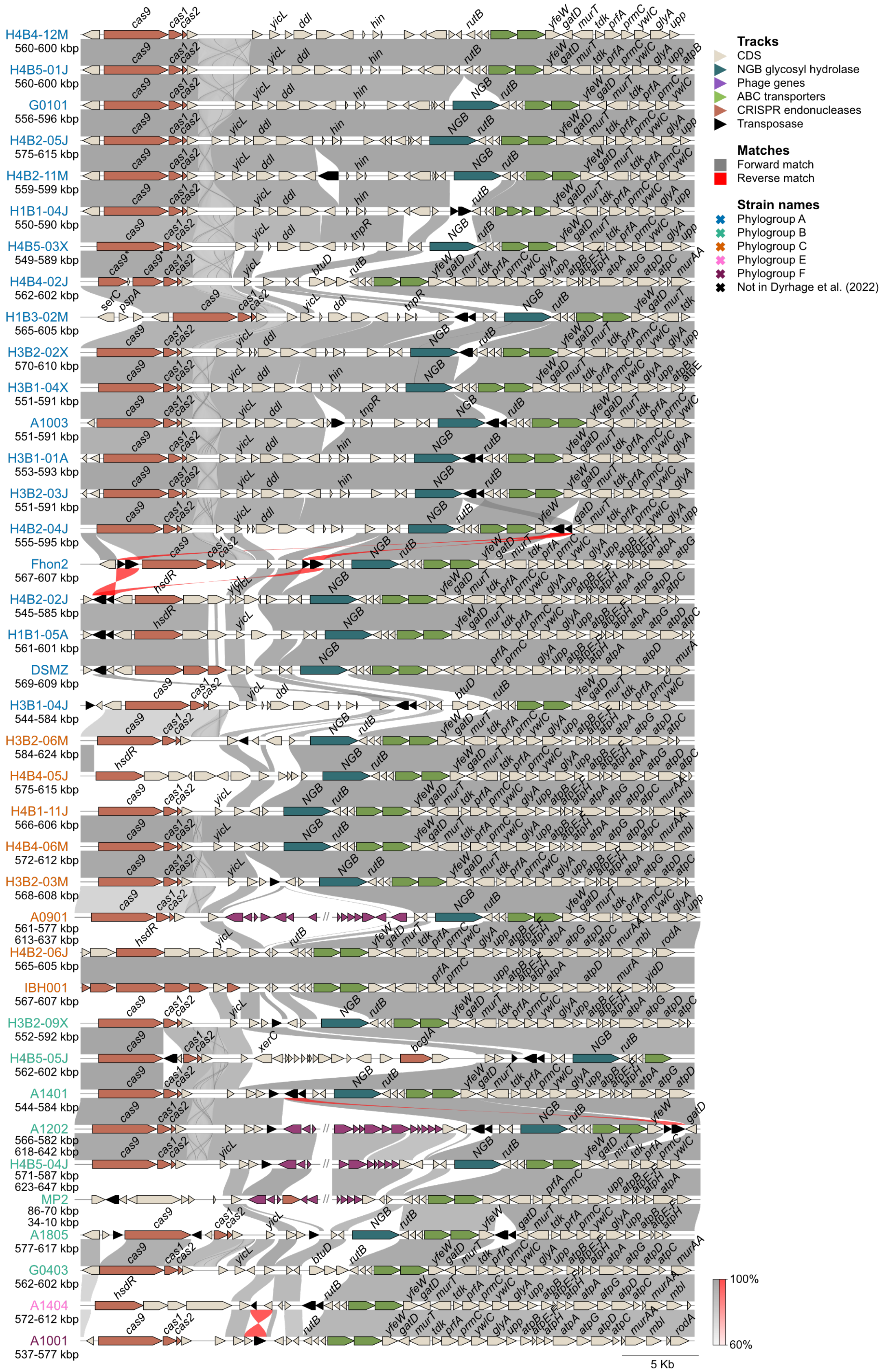

H4B4-12M
560-600 kbp
H4B5-01J
560-600 kbp
G0101
556-596 kbp
H4B2-05J
575-615 kbp
H4B2-11M
559-599 kbp
H1B1-04J
550-590 kbp
H4B5-03X
549-589 kbp
H4B4-02J
562-602 kbp
H1B3-02M
565-605 kbp
H3B2-02X
570-610 kbp
H3B1-04X
551-591 kbp
A1003
551-591 kbp
H3B1-01A
553-593 kbp
H3B2-03J
551-591 kbp
H4B2-04J
555-595 kbp
Fhon2
567-607 kbp
H4B2-02J
545-585 kbp
H1B1-05A
561-601 kbp
DSMZ
569-609 kbp
H3B1-04J
544-584 kbp
H3B2-06M
584-624 kbp
H4B4-05J
575-615 kbp
H4B1-11J
566-606 kbp
H4B4-06M
572-612 kbp
H3B2-03M
568-608 kbp
A0901
561-577 kbp
613-637 kbp
H4B2-06J
565-605 kbp
IBH001
567-607 kbp
H3B2-09X
552-592 kbp
H4B5-05J
562-602 kbp
A1401
544-584 kbp
A1202
566-582 kbp
618-642 kbp
H4B5-04J
571-587 kbp
623-647 kbp
MP2
86-70 kbp
34-10 kbp
A1805
577-617 kbp
G0403
562-602 kbp
A1404
572-612 kbp
A1001
537-577 kbp
cas9
cas1
cas2
yicL
ddl
hin
rutB
NGB
yfeW
gatD
murT
tdk
prfA
prmC
ywlC
glyA
upp
atpB
atpE
atpF
atpH
atpA
atpG
atpD
atpC
murAA
mbl
rodA
yidD
tnpR
btuD
serC
pspA
hsdR
xerC
bcglA
Tracks
CDS
NGB glycosyl hydrolase
Phage genes
ABC transporters
CRISPR endonucleases
Transposase
Matches
Forward match
Reverse match
Strain names
Phylogroup A
Phylogroup B
Phylogroup C
Phylogroup E
Phylogroup F
Not in Dyrhage et al. (2022)
100%
60%
5 Kb


Figure S5

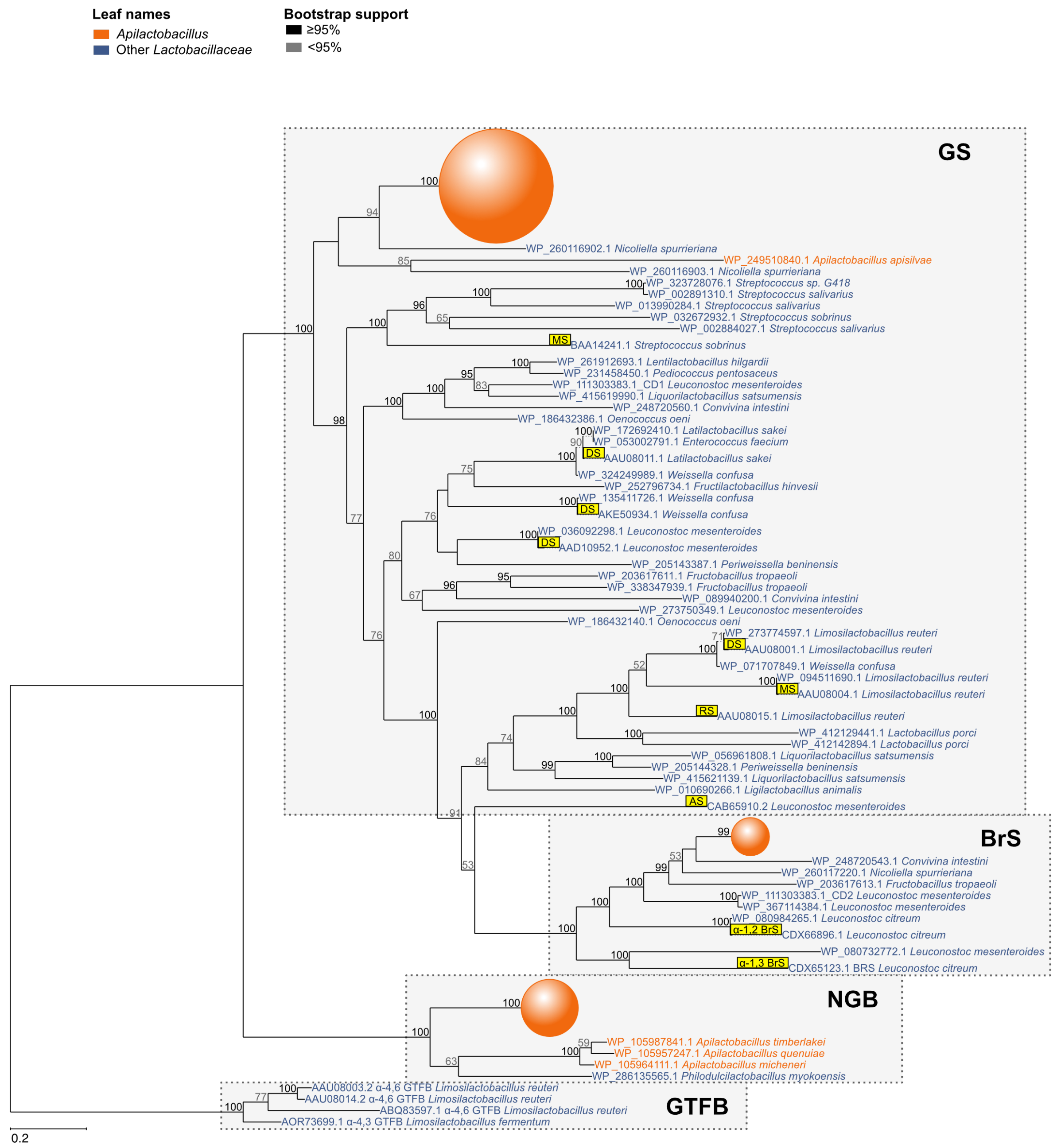
Leaf names
Apilactobacillus
Other Lactobacillaceae
Bootstrap support
≥95%
<95%
GS
WP_260116902.1 Nicoliella spurrieriana
WP_249510840.1 Apilactobacillus apisilvae
WP_260116903.1 Nicoliella spurrieriana
WP_323728076.1 Streptococcus sp. G418
WP_002891310.1 Streptococcus salivarius
WP_013990284.1 Streptococcus salivarius
WP_032672932.1 Streptococcus sobrinus
WP_002884027.1 Streptococcus salivarius
MS
BAA14241.1 Streptococcus sobrinus
WP_261912693.1 Lentilactobacillus hilgardii
WP_231458450.1 Pediococcus pentosaceus
WP_111303383.1_CD1 Leuconostoc mesenteroides
WP_415619990.1 Liquorilactobacillus satsumensis
WP_248720560.1 Convivina intestini
WP_186432386.1 Oenococcus oeni
WP_172692410.1 Latilactobacillus sakei
WP_053002791.1 Enterococcus faecium
DS
AAU08011.1 Latilactobacillus sakei
WP_324249989.1 Weissella confusa
WP_252796734.1 Fructilactobacillus hinvesii
WP_135411726.1 Weissella confusa
DS
AKE50934.1 Weissella confusa
WP_036092298.1 Leuconostoc mesenteroides
DS
AAD10952.1 Leuconostoc mesenteroides
WP_205143387.1 Periweissella beninensis
WP_203617611.1 Fructobacillus tropaeoli
WP_338347939.1 Fructobacillus tropaeoli
WP_089940200.1 Convivina intestini
WP_273750349.1 Leuconostoc mesenteroides
WP_186432140.1 Oenococcus oeni
WP_273774597.1 Limosilactobacillus reuteri
DS
AAU08001.1 Limosilactobacillus reuteri
WP_071707849.1 Weissella confusa
WP_094511690.1 Limosilactobacillus reuteri
MS
AAU08004.1 Limosilactobacillus reuteri
RS
AAU08015.1 Limosilactobacillus reuteri
WP_412129441.1 Lactobacillus porci
WP_412142894.1 Lactobacillus porci
WP_056961808.1 Liquorilactobacillus satsumensis
WP_205144328.1 Periweissella beninensis
WP_415621139.1 Liquorilactobacillus satsumensis
WP_010690266.1 Ligilactobacillus animalis
AS
CAB65910.2 Leuconostoc mesenteroides
BrS
WP_248720543.1 Convivina intestini
WP_260117220.1 Nicoliella spurrieriana
WP_203617613.1 Fructobacillus tropaeoli
WP_111303383.1_CD2 Leuconostoc mesenteroides
WP_367114384.1 Leuconostoc mesenteroides
WP_080984265.1 Leuconostoc citreum
α-1,2 BrS
CDX66896.1 Leuconostoc citreum
WP_080732772.1 Leuconostoc mesenteroides
α-1,3 BrS
CDX65123.1 BRS Leuconostoc citreum
NGB
WP_105987841.1 Apilactobacillus timberlakei
WP_105957247.1 Apilactobacillus quenuiae
WP_105964111.1 Apilactobacillus micheneri
WP_286135565.1 Philodulcilactobacillus myokoensis
GTFB
AAU08003.2 α-4,6 GTFB Limosilactobacillus reuteri
AAU08014.2 α-4,6 GTFB Limosilactobacillus reuteri
ABQ83597.1 α-4,6 GTFB Limosilactobacillus reuteri
AOR73699.1 α-4,3 GTFB Limosilactobacillus fermentum
0.2

Figure S6

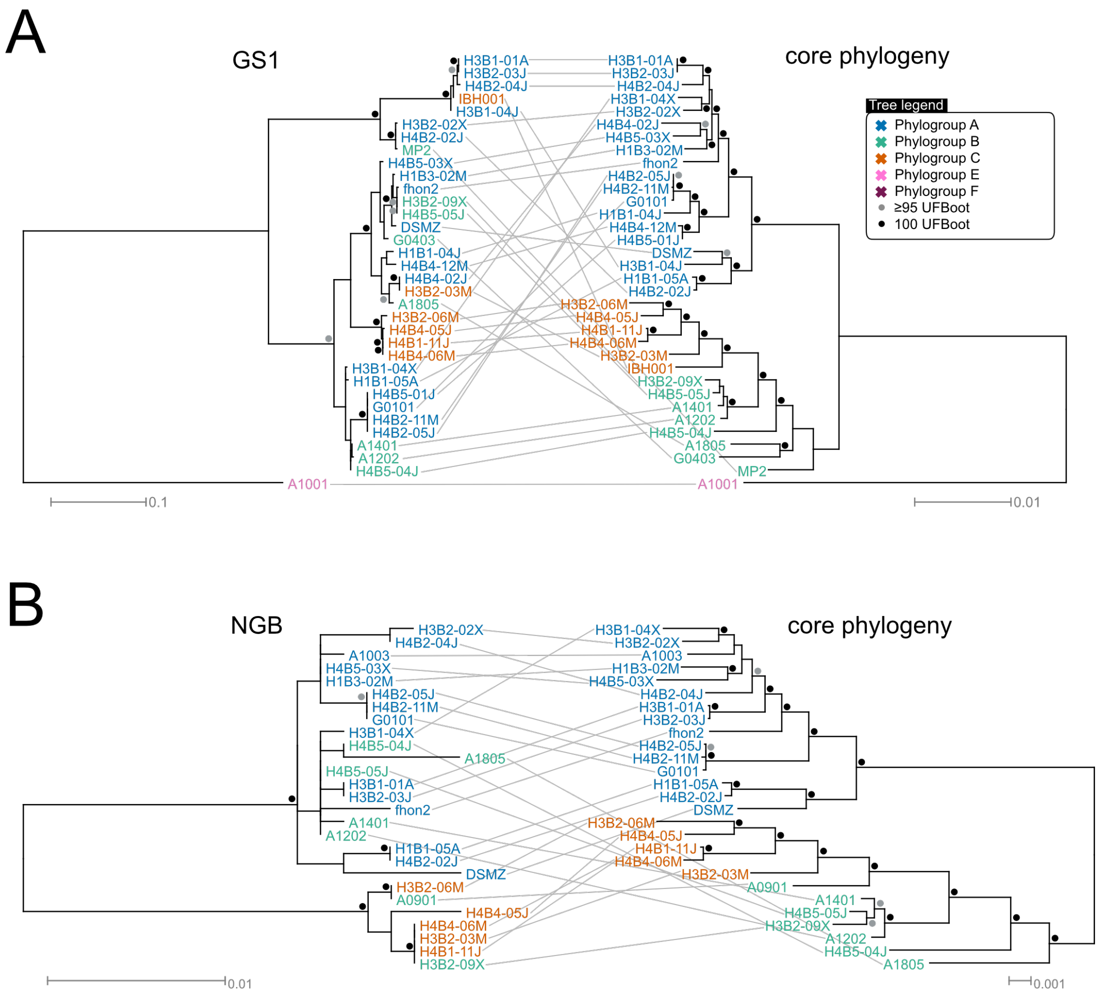
A
GS1
core phylogeny
H3B1-01A
H3B2-03J
H4B2-04J
IBH001
H3B1-04J
H3B2-02X
H4B2-02J
MP2
H4B5-03X
H1B3-02M
fhon2
H3B2-09X
H4B5-05J
DSMZ
G0403
H1B1-04J
H4B4-12M
H4B4-02J
H3B2-03M
A1805
H3B2-06M
H4B4-05J
H4B1-11J
H4B4-06M
H3B1-04X
H1B1-05A
H4B5-01J
G0101
H4B2-11M
H4B2-05J
A1401
A1202
H4B5-04J
A1001
0.1
0.01
Tree legend
Phylogroup A
Phylogroup B
Phylogroup C
Phylogroup E
Phylogroup F
≥95 UFBoot
100 UFBoot
B
NGB
core phylogeny
A1003
A0901
0.01
0.001

Figure S7A-B

C

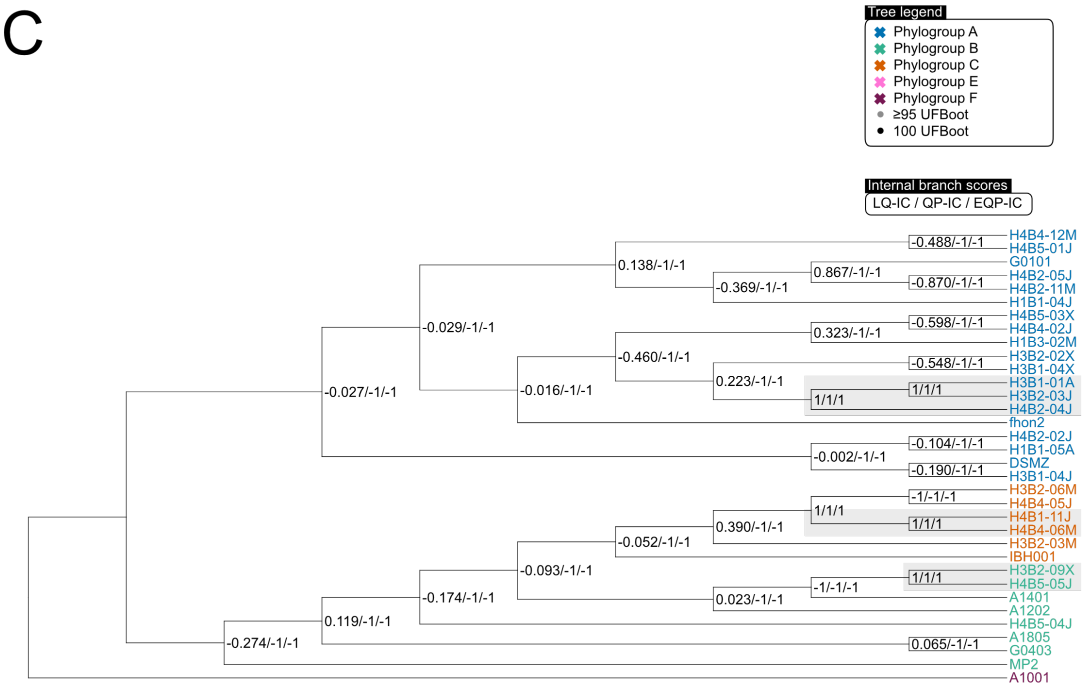


D

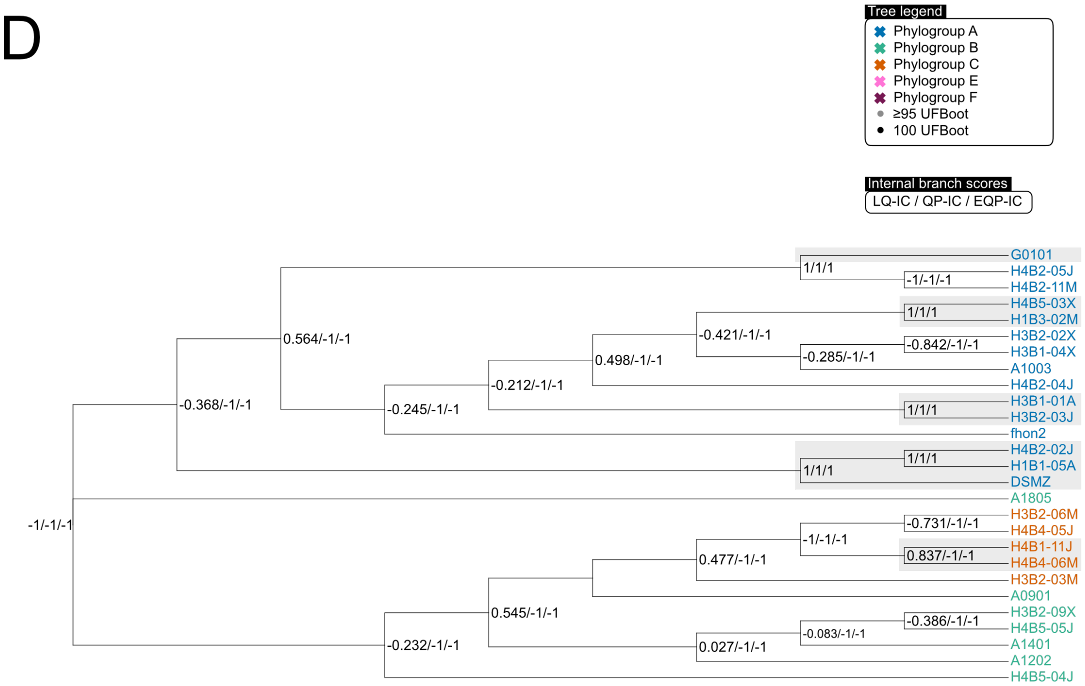


Figure S7C-D

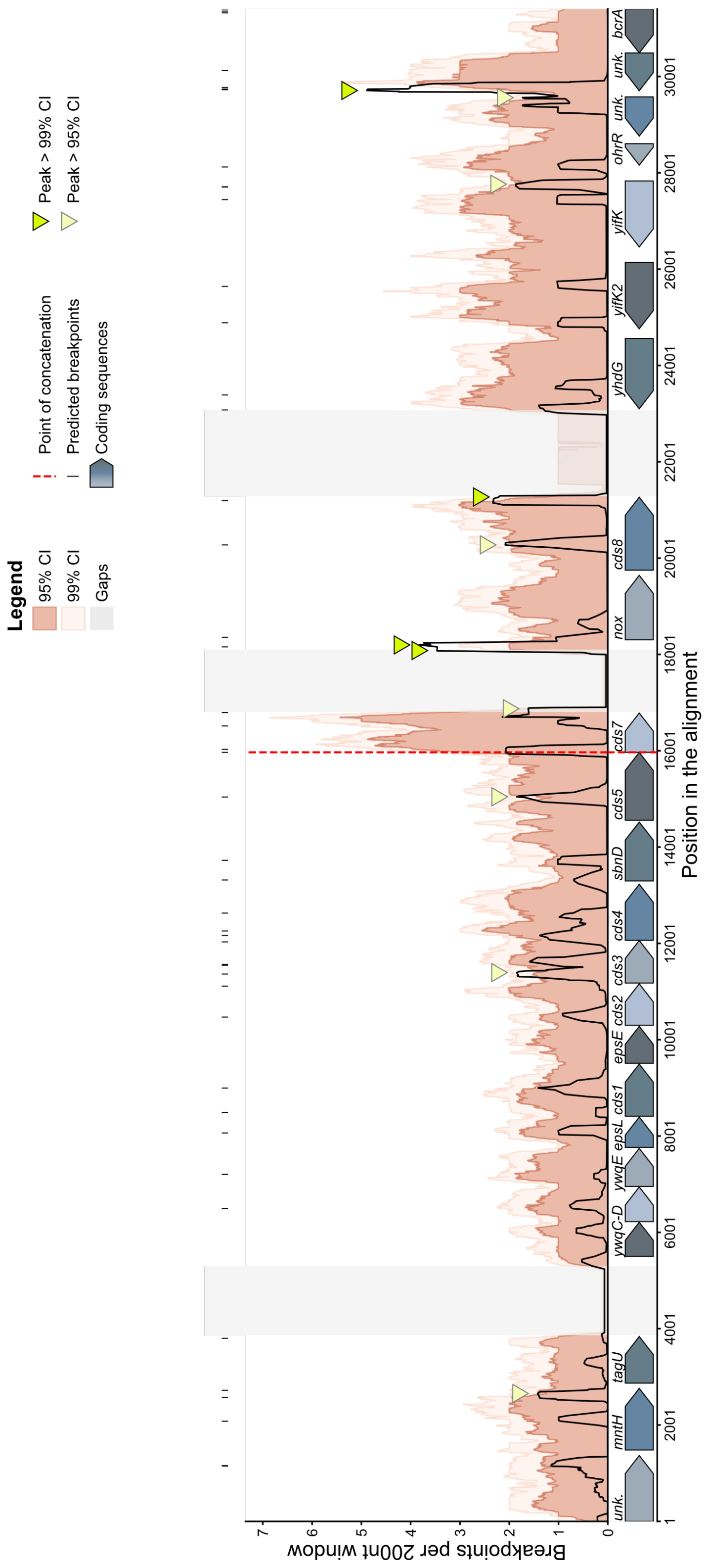
Legend
95% CI
99% CI
Gaps
Point of concatenation
Predicted breakpoints
Coding sequences
Peak > 99% CI
Peak > 95% CI
Breakpoints per 200nt window
Position in the alignment
unk.
mntH
tagU
ywqC-D
ywqE
epsL
cds1
epsE
cds2
cds3
cds4
sbnD
cds5
cds7
nox
cds8
yhdG
yifK2
yifK
ohrR
unk.
unk.
bcrA

Figure S8

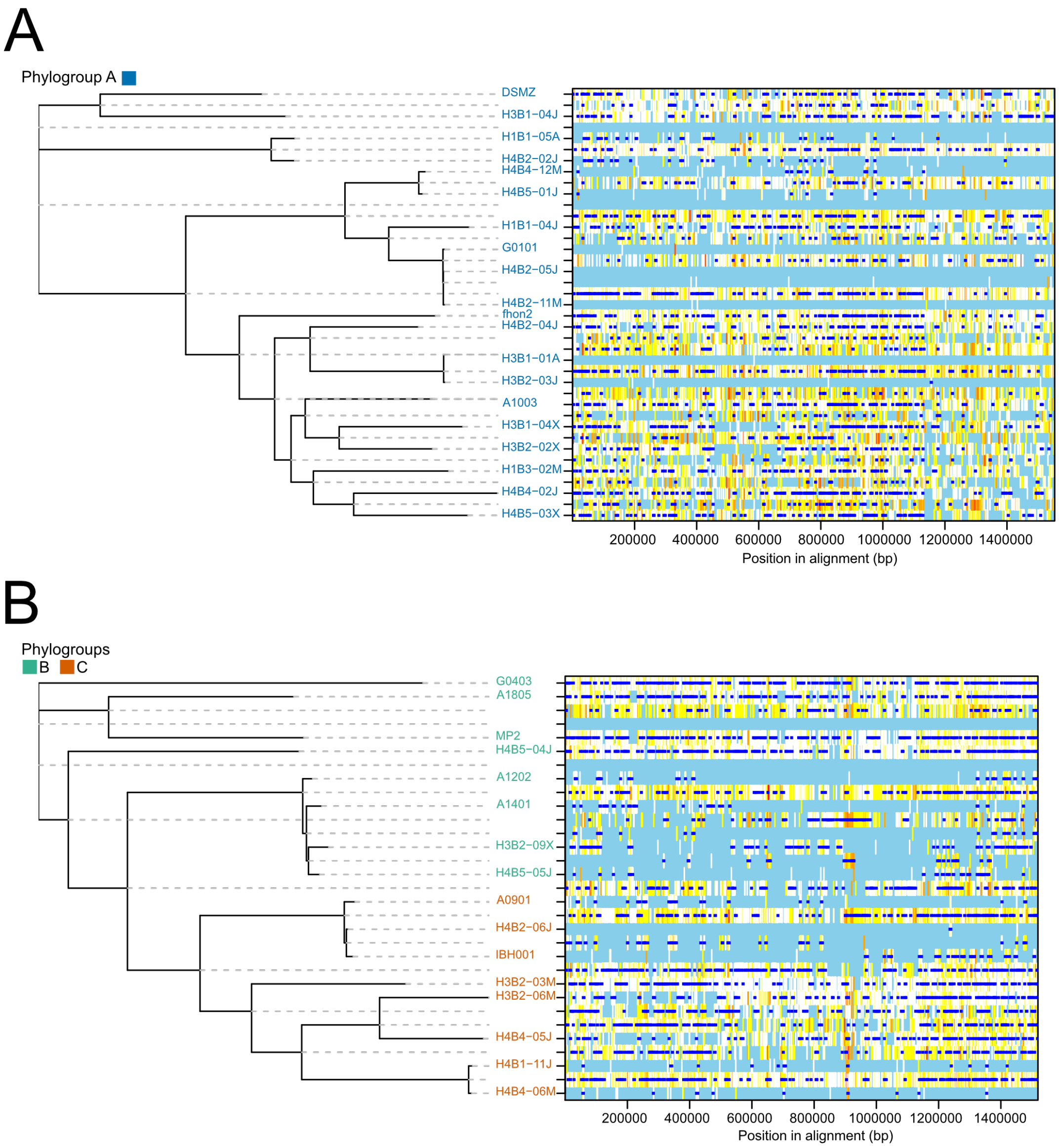
A
Phylogroup A
DSMZ
H3B1−04J
H1B1−05A
H4B2−02J
H4B4−12M
H4B5−01J
H1B1−04J
G0101
H4B2−05J
H4B2−11M
fhon2
H4B2−04J
H3B1−01A
H3B2−03J
A1003
H3B1−04X
H3B2−02X
H1B3−02M
H4B4−02J
H4B5−03X
200000
400000
600000
800000
1000000
1200000
1400000
Position in alignment (bp)
B
Phylogroups
B
C
G0403
A1805
MP2
H4B5−04J
A1202
A1401
H3B2−09X
H4B5−05J
A0901
H4B2−06J
IBH001
H3B2−03M
H3B2−06M
H4B4−05J
H4B1−11J
H4B4−06M
200000
400000
600000
800000
1000000
1200000
1400000
Position in alignment (bp)

Figure S9

## Supplementary Figure Legends

**Figure S1. Sanger sequencing chromatograms of the PCR products of primer pairs targeting GH genes** A) A1401_12760-70 (GS2 and BrS segments), B) H3B111M_12570-80 (BrS), C) FHON2_13560-70 (GS2), D) H3B209X_13360-70 (GS2), E) G0102_12710-20 (GS1). Putative stop codons are highlighted in grey, and differences with the original sequence are highlighted in red. R = ruler, - = negative control.

**Figure S2**. **Phylogenetic relationships of GH70 and GH32 domains.** Phylogenetic trees were inferred from the amino acid alignments of the A) 907 core proteins, B) 97 GH70 domains plus four outgroups and C) 25 GH32 domains in 38 representative strains of *A. kunkeei*. Strain names and locus tags are colored by phylogroup designations according to the core protein phylogeny by Dyrhage et al. (2022). The catalytic activities of protein domains with experimentally confirmed functions are indicated in the yellow boxes. Proteins annotated as GTFB in *Limosilactobacillus reuteri* and *Limosilactobacillus fermentum* were used as outgroups in the GH70 domain phylogeny. The trees were inferred with maximum likelihood methods. Numbers on nodes show bootstrap support values ≥50.

**Figure S3**. **Possible domain organizations of the GH70 and GH32 family proteins.** Organization of domains and sequence repeats in a subset of A) GH70 and B) GH32 enzymes with and without glucan-binding repeats in the 38 reference *A. kunkeei* strains. All domains and repeats are based on InterProScan predictions, except for signal peptides, which are based on SignalP6 (slow mode) predictions; glucan-binding domains, which are based on AlphaFold predictions, and serine-rich repeats, which are based on protein sequence. GS = glucansucrases, BrS = branching sucrases, NGB = non-glucan binding GH70, PTL = proteins with the N-terminal segment of a GH70 domain, S1-3 = GH32 subtypes, of which S3 lacks glucan-binding domains.

**Figure S4. Pairwise BLASTN comparisons of a 40 kb segment of the *A. kunkeei* genome with the genes coding for GH70 and GH32 enzymes.** The order of strains is based on the core protein phylogeny shown in Figure S2A. Strain names are colored by phylogroup designations. Forward BLASTN matches are colored in grey and reverse matches, in red. GH70 and GH32 are colored depending on the subtype, and transposons are colored in black.

**Figure S5**. **Pairwise BLASTN comparisons of a 40 kb segment of the *A. kunkeei* genome with the genes coding for the NGB enzymes.** The order of strains is based on the core protein phylogeny shown in Figure S2A. Strain names are colored by phylogroup designations. Forward BLASTN matches are colored in grey and reverse matches, in red. NGB is colored in dark blue-green, phage genes in purple, ABC transporters in green, CRISPR genes in brown and transposons in black.

**Figure S6**. **Phylogenetic relationships of GH70 domains in bacteria.** Phylogenetic trees were inferred with the maximum likelihood method from the amino acid alignments of GH70 domains in members of the *Lactobacillaceae, Streptococcaceae* and *Enterococcaceae*. The clades containing GSs, BrSs and NGBs in the reference *A. kunkeei* strains and *A. apinorum* are schematically indicated by circles colored in orange. Species names and gene IDs are colored by family designations. The catalytic activities of protein domains with experimentally confirmed functions are indicated by AS (alternansucrase), DS (dextransucrase), MS (mutansucrase), RS (reuteransucrase) or BrS labels, all marked in yellow. Proteins annotated

as GTFB in *Limosilactobacillus reuteri* and *Limosilactobacillus fermentum* were used as outgroups. The subtypes within which the domains cluster (GS, BrS, NGB or GTFB) are indicated by dotted rectangles. Numbers on nodes show bootstrap support values ≥50.

**Figure S7. Comparison of GH70 domain tree topologies.** Comparisons of A, B) tree topologies and C, D) quartet-based internode certainty scores for GH70 and core proteins. Phylogenetic trees and were inferred based on the amino acid alignments of the A) GS1 and B) NGB domains in the two subsets of *A. kunkeei* strains that contain genes for these proteins. The tree topologies were compared to the topologies obtained from phylogenetic inferences based on 907 core proteins in the two subsets of *A. kunkeei* strains, respectively. The trees were inferred with the maximum likelihood method. Bootstrap support values of 100% and ≥ 95% are shown as black and grey dots, respectively. Quartet-based internode certainty score comparisons of the core protein phylogenies and the C) GS1 and D) NGB domain phylogenies. The branch values display the lowest quartet internode certainty (LQ-IC), the quadripartition internode certainty (QP-IC) and the extended quadripartition internode certainty (EQP-IC) scores, where values close to 1 indicates support of the internal branch, values close to 0 indicate high levels of incongruence and values close to -1 contest it. Branches with LQ-IC scores close to 1 are highlighted. The GH70 and core protein input phylogenies were unrooted in the QuartetScore analysis, and the resulting trees were midpoint rooted for visualization purposes. Strain names and locus tags are colored by phylogroup designations.

**Figure S8. Recombination breakpoint plot, showing the genes in the genomic region for GH70 and GH32 family proteins.** The lines at the top of the plot represent predicted breakpoints. The dashed red line represents the join point between the two alignments. Gaps are shown as grey rectangles. The continuous black line represents the number of predicted breakpoints per 200 nt windows along the alignment. The two shaded areas behind the black line represent the confidence intervals (CIs), where the strongly-shaded area represents the 95% CI and the faintly-shaded area represents the 99% CI. Green triangles represent parts of the alignment where the number of predicted breakpoints is above the 95% CI, if less saturated, or the 99% CI, if more saturated. The coding sequences along the alignment are shown at the bottom.

**Figure S9. ClonalFrameML plot showing recombination along the *A. kunkeei* chromosome** for A) phylogroup A and B) phylogroups B-C. Dark blue = predicted recombination. Light blue = no change. White, yellow and orange represent the degree of homoplasy, with white being the lowest and orange, the highest.
